# A machine learning approach to the digitalization of bank customers: Evidence from random and causal forests

**DOI:** 10.1371/journal.pone.0240362

**Published:** 2020-10-28

**Authors:** Santiago Carbo-Valverde, Pedro Cuadros-Solas, Francisco Rodríguez-Fernández

**Affiliations:** 1 University of Granada, Granada, Spain; 2 Bangor University, Hen Goleg, Bangor, United Kingdom; 3 Funcas, Madrid, Spain; 4 CUNEF, Madrid, Spain; Shandong University of Science and Technology, CHINA

## Abstract

Understanding the digital jump of bank customers is key to design strategies to bring on board and keep online users, as well as to explain the increasing competition from new providers of financial services (such as BigTech and FinTech). This paper employs a machine learning approach to examine the digitalization process of bank customers using a comprehensive consumer finance survey. By employing a set of algorithms (random forests, conditional inference trees and causal forests) this paper identities the features predicting bank customers’ digitalization process, illustrates the sequence of consumers’ decision-making actions and explores the existence of causal relationships in the digitalization process. Random forests are found to provide the highest performance–they accurately predict 88.41% of bank customers’ online banking adoption and usage decisions. We find that the adoption of digital banking services begins with information-based services (e.g., checking account balance), conditional on the awareness of the range of online services by customers, and then is followed by transactional services (e.g., online/mobile money transfer). The diversification of the use of online channels is explained by the consciousness about the range of services available and the safety perception. A certain degree of complementarity between bank and non-bank digital channels is also found. The treatment effect estimations of the causal forest algorithms confirm causality of the identified explanatory factors. These results suggest that banks should address the digital transformation of their customers by segmenting them according to their revealed preferences and offering them personalized digital services. Additionally, policymakers should promote financial digitalization, designing policies oriented towards making consumers aware of the range of online services available.

## 1. Introduction

At the end of 2019, 53.6% of the global population, or 4.1 billion people, used online digital devices, according to Information and Communications Technology (ICT). Digitalization is changing the shape of many industries and the way companies and clients interact. This digital revolution has been particularly relevant in the banking industry where the use of digital banking (online and mobile) has become one of the most strategic channels used by bank customers. The Organization for Economic Co-operation and Development (OECD) has identified some of the core properties and crosscutting effects of the digital transformation [1] as the most important business challenge currently underway. Furthermore, the OECD recognizes banking as one of the sectors where such transformation is more relevant in economic, organizational, and social terms.

On the supply side, financial institutions have gradually reacted to these changes. Banks are particularly sensitive to the transformation of information systems, the treatment of personal data, and the emergence of new (fully digital) competitors and delivery channels. Despite incorporating online distribution channels two decades ago, and in spite of the renewed digitalization wave, banks continue to develop more information and systems-oriented business models. Digitalization is not only focused on cost savings, but also includes process improvements to enhance customer experiences [2–5]. This effort is driven by both rival precedence [6, 7] and changes in demand [8].

A large number of studies on banking organization and technology have addressed the adoption of the most basic electronic banking services developed over the last few decades including debit and credit cards and more recently online banking (although partially covered). Prior literature has revealed a variety of mechanisms—motivations, attitudes, behavioral intention, social systems, and associations—involved in technology adoption. These studies have found that perceived security, usefulness, quality, and convenience drive consumer adoption of online services [9–14]. However, the relevance of each of these factors depends on the stage of the adoption. This is an important lesson for new digital services given the heterogeneous penetration they have both geographically and demographically [15]. This is particularly relevant considering that socio-demographic characteristics—age, gender, income, and location—[11, 16, 17], cultural characteristics [18], and customer experience (with other products with varying levels of technological sophistication) are strongly related to the demand for online banking services [19].

However, while the initial adoption of digital services could be examined using standard parametric statistical methods, examining customers’ digital journey is more complex. Digitalization is a challenging endeavor where several factors drive digitalization decisions [5, 20]. Machine learning methods have emerged as powerful tools for data mining [21–24]. Instead of being limited to making strong assumptions about the structure of the data, machine learning allows researchers to identify and display complex patterns in a data-driven form [25]. In this sense, a machine learning approach is gaining ground in examining consumer behavior such as consumer preferences for technology products Chen, Honda, & Yang [26], travel choices [27] or to model consumer response [21].

This paper aims to benefit from the advantages of following a machine learning approach in order to examine the bank customers’ digitalization process. The use of machine and causal machine algorithms in our research context allows us to reveal the process that individuals follow to make their financial digitalization choices. Unlike prior studies, we are not focused on a single dimension of the digitalization process but on several dimensions (adoption, diversity of use and bank and non-bank’s payment choices).

Methodologically, instead of ex-ante selecting a machine learning technique, we consider a number of machine learning techniques that have proved their value in this field: random forest, extreme gradient boosting, k-nearest neighbor, support vector machine, Bayesian networks and extreme learning machine (see among others [28–32]). After selecting the machine learning with the best performance (in terms of predicted accuracy) we use this algorithm to identify the main features predicting bank customers’ digitalization process. Then, we build a set of classification trees to illustrate the sequence of consumers’ decision-making, and, finally, we make use of causal forests (a causal machine learning technique) to estimate the existence of causal relationships in the digitalization process.

The empirical analysis relies on extensive data collected from a survey—following the structure of the Survey of Consumer Payment Choice (SCPC) [33]—about digital banking and payment services responded by 3,005 consumers between the ages of 18 and 75. This dataset allows us to explore financial digitalization in a developed country with deep internet penetration (84.6% of adults are internet users), a highly banked population (97.2% of adults have a bank account), and a growing use of electronic banking among consumers (62% of the sample individuals are e-banking users to some extent, although the degree and scope of the adoption varies substantially across individuals), according to OECD, World Bank and GlobalWeb data.

By way of preview, we find that the random forest algorithm achieves the best performance in terms of accuracy to predict bank customers’ digitalization. This algorithm -coupled with the classification trees- reveals that bank customers need to become familiar with the information content of digital services before they begin to make financial transactions. Going digital begins with information-based services and is then followed by transactional services. Customers check their bank balances, make inquiries, and explore the possibilities of the digital channels before making payments, transferring money, or engaging in other transactional services. As for the scope of digitalization, the perceived safety of digital bank services by consumers becomes a critical filter for consumers’ diversified use of digital bank services. However, there appear to be notable exceptions. In the case of mobile banking, for example, even if perceived safety influences consumers’ adoption decisions, the speed and ease of use of the device appear to be more decisive. The efficiency of this service contrasts with the adoption process of more traditional and more established bank services such as credit and debit cards, which are used on a regular basis only when they are perceived as safe and relatively costless. Moreover, consumers adopt other non-bank digital financial services (e.g., Amazon or PayPal) only after they have already become frequent and diversified digital bank customers. These results are also confirmed when using the extreme gradient boosting algorithm and plotting a Bayesian network for each of the dimensions considered. Causal forests reveal that checking online balances has the largest effect on adopting online banking, while making money transfers with a smartphone seems to be relatively more important to become a diversified mobile banking customer. Regarding the use of bank payment methods, we find that the perception of safety has the largest impact on using credit cards while the perception of cost and convenience have the largest impact on paying with debit cards.

These results seem to have relevant business implications for the banking industry when designing strategies to bring on board and keep digital users (e.g., offer digital services focused on satisfying customers’ needs), to face the increasing competition in the payment sector by BigTech and FinTech (e.g. link payments experiences with social media) or to succeed with their digitalization programs (e.g. segmenting customers). Moreover, these results are also valuable for policymakers to design efficient measures to promote financial digitalization.

The remainder of the paper is organized as follows: Section 2 reviews the related literature; Section 3 describes the dataset and the methodology employed; Section 4 discusses the main empirical results; Section 5 addresses the causal impact using causal forests; Section 6 shows the consistency of the findings over alternative supply-side explanations and presents the implications, limitations, and scope for future research; and Section 7 concludes.

## 2. Related works

The main relevant studies related to financial technology adoption in the digital age refer to firm management and information systems. A number of theories aim to explain the evolution of these new technologies and the interaction between the consumer and the firm. Among them, the technology acceptance model (TAM) [34] and its latter versions (TAM2 and TAM3) have become popular for explaining how people accept and adopt new technology in the context of banking. The TAM model, which is based on the theory of reasonable action (TRA) [35] and the theory of planned behavior (TPB) (Ajzen [36, 37]), suggests that technological adoption depends on customers’ perception of the utility and ease of use of the technology. Other theories such as the diffusion of innovations (DIT) [38], the task-technology fit (TTF) [39], the unified theory of acceptance and use of technology (UTAUT) [40], and the technology resistance theory (TRT) [41] have complemented the drivers of online adoption. These theories have thereby given prominence to a number of technological components of the service and not just to consumers’ perceptions. However, as it has recently been argued, those factors explored by the existing literature on information systems may not be sufficient to explain banking digitalization [20, 42].

From an empirical standpoint, prior studies on customers’ perceptions have identified the main factors that explain the adoption and utilization of online banking. These include security [9, 10, 13, 43], ease of use [12–14, 44, 45], convenience [12, 13], and cost [11, 46]. Overall, consumers use e-banking services when they perceive them as safe, useful, convenient, and relatively costless. As for the relative importance of these factors, Hoehle et al. [10] have surveyed the literature and concluded that security is a major determinant of consumers’ use of e-banking services. Additionally, many of these studies highlight that a range of socio-demographic characteristics [11, 16, 17] and cultural characteristics [18] also influence the adoption of online banking services. Specifically, young people who have a higher income and live in areas of high internet penetration [11, 47, 48] are prone to using online services. However, as Montazemi and Qahri-Saremi [15] have highlighted, the importance of these socio-demographic factors depends on the stage of the adoption of online banking services within each market segment or jurisdiction. Moreover, Szopiński [19] has found that having other banking products such as mortgages and credit cards also has a significant influence on consumers’ use of online banking services.

Closely related to online banking, studies on mobile banking adoption have also recently emerged. The empirical and theoretical approaches in these studies are similar to those to online banking [49–53]. The results of these studies suggest that age is the most decisive factor in mobile banking adoption. However, other determinants such as trust in the device, security, and cost have also been reported to strongly influence the adoption of mobile payments [54].

Our paper aims to offer a twofold contribution to the existing literature on bank customers’ digitalization. First, by employing a machine learning approach, it reveals the patterns driving the digitalization process. Second, unlike prior studies we do not focus on a single dimension of digitalization. We explore the digital journey of bank customers by examining a number of dimensions (adoption, diversity of use and bank and non-bank’s payment choices) to provide a more complete picture of the digitalization process.

## 3. Data

### 3.1 The survey

The primary data for this study were collected from a consumer survey that was specifically conducted for this research by IMOP Insights during November and December 2016. The survey participants—a population of Spanish consumers between the ages of 18 and 75—were asked about their digital preferences and in particular about those related to banking and payment services. The survey followed the structure of the Survey of Consumer Payment Choice (SCPC) originally conducted by the Federal Reserve Bank of Boston and it is currently conducted by the Federal Reserve Bank of Atlanta. However, our survey incorporated comprehensive information about consumers’ digital preferences and not just about payment services. Controlled quotas for a representative sample of the population were established based on age, sex, and location. The survey was conducted via telephone interviews and resulted in a sample size of 3,005 consumers. The human participation in this study is simply the voluntary participation of subjects in a telephone survey conducted with all the legal and sociological guarantees. The consent of all the survey participants was informed before conducting the questionnaire and this consent was documented as part of the recorded telephone survey. Data were analyzed anonymously by the authors. S1 Appendix in S1 File offers detailed information about the survey and the data collection process and all the variables extracted from the survey questionnaire.

Spain seems to be a good laboratory for this study because it has overcome the initial implementation phase of electronic banking and ranks third in the world for annual growth in mobile banking adoption, according to OECD statistics. The penetration of online banking and the general level of financial digitalization in Spanish society are similar to those in other developed economies. Consequently, the main findings—with the necessary caveats—could likely to be extrapolated to other jurisdictions or would at least be useful for informing other research in different countries. Table 1 illustrates the sample demographics. The representativeness of the survey data is assured by comparing the sample breakdown with the Spanish National Statistics Institute (INE).

**Table 1:** Sample demographics.

	n	%	Official Statistics (%)
**Gender**			
Male	1493	49.7	48.7
Female	1512	50.3	51.3
**Age (years)**			
18–24	282	9.4	10.4
25–34	498	16.6	14.1
35–44	686	22.8	19.8
45–54	631	21.0	18.7
55–64	500	16.6	14.9
65–75	408	13.6	14.1
**Habitat (inhabitants)**			
0–10000	637	21.2	20.6
10001–50000	806	26.8	26.9
50001–200000	696	23.2	18.7
> 200000	866	28.8	33.8
**N° People at home**			
1 person	644	21.4	19.6
Two people	850	28.3	24.2
Three people	757	25.2	25.2
More than three people	754	25.1	31.0
**Employment situation**			
Working	1815	60.4	55.2
Pensioner/retired	500	16.6	24.9
Unemployed	338	11.2	9.1
Student	193	6.4	10.9
Unpaid domestic work	159	5.3	
**Sample size**	3005	100	

Table 1 provides a breakdown of the 3,005 surveyed participants by gender, age, habitat, number of people living at home and employment situation.

### 3.2 Descriptive statistics

**Fig 1:**
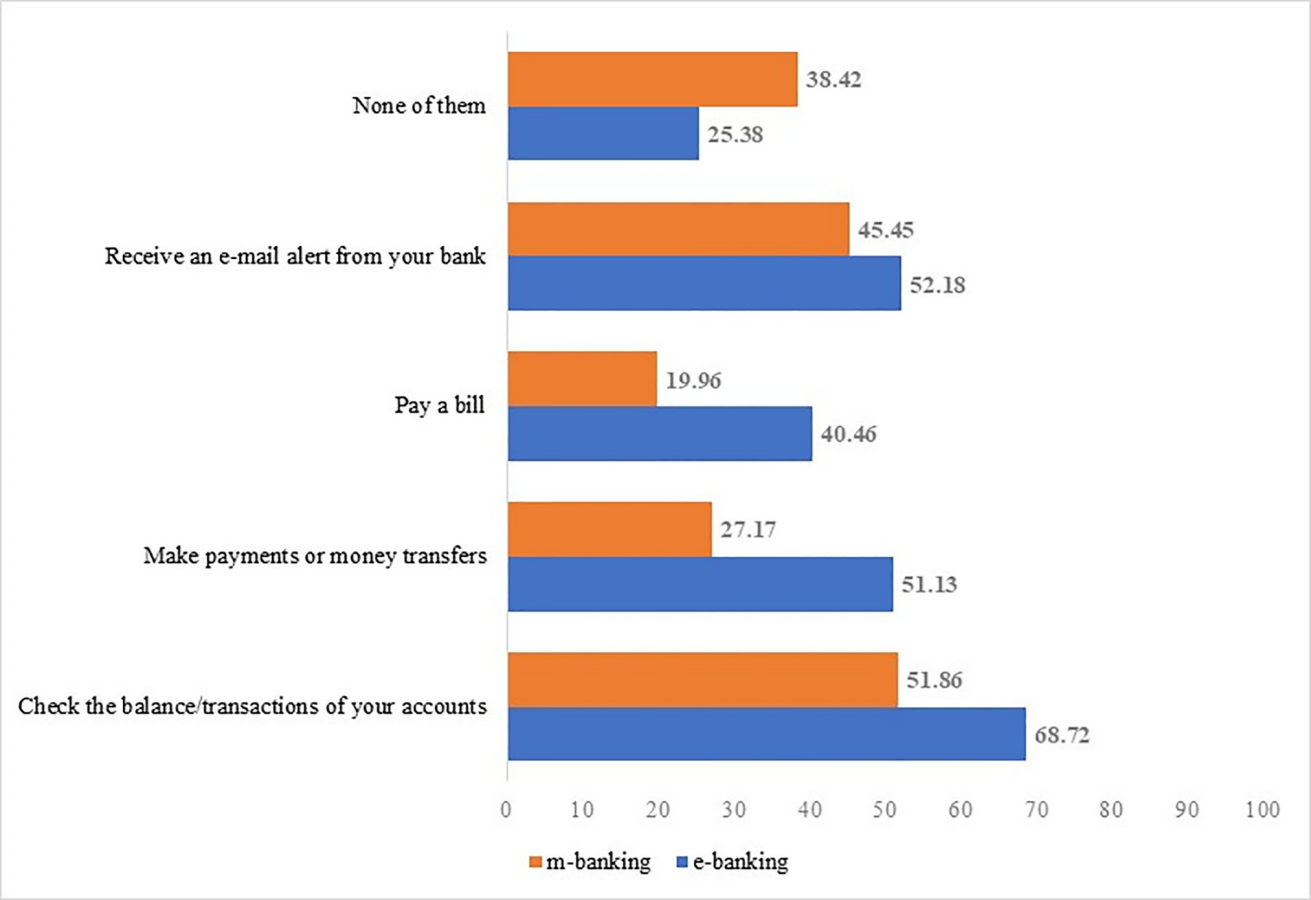
Degree of financial digitalization: Financial online activities (% of internet users). Fig 1 illustrates the degree to which consumers use various financial services: receiving emails, paying bills, making payments and checking the account balances.

Fig 1 illustrates the degree to which consumers use various financial services. On average, each banking client has two bank accounts and operates with more than one entity. It is worth noting that while 79.6% of respondents have an online bank account, only 13% are exclusively online users. Regarding the type of financial activities conducted online, internet users reported accessing online banking services to check account balances (68.72% of respondents), to receive online communications from their bank (52.18%), and to make payments or transfer money (51.13%). In the case of mobile banking, the activities lean even more toward checking and communication rather than transactional services.

Fig 19 also provides some descriptive statistics on consumers’ perceptions and on the adoption of non-banking services and social networks by gender, age, and employment status. 91.3% of participants are frequent internet users. In terms of digital equipment, 74.8% of them reported having a laptop, 98.5% reported having a mobile phone and 46.8% reported having a tablet. In any event, the figures suggest that Spanish consumers have attained a medium-high degree of digitalization and a medium degree of financial digitalization. 79.5% of participants have an online bank account. In general, it seems that adults under the age of 45 (working or studying) are the most digitalized. It does not seem to be a gender gap in terms of financial digitalization. More significant differences emerge by employment status as working people tend to be more digitalized than the unemployed. In terms of perceptions, while most of the people perceive online and mobile banking as having a low or very low cost and safe or very safe, this percentage is smaller for those above 65 years. Finally, as expected, young people and users of social media are also more frequent adopters. 50.7% of young people (18–24 years old) have a non-bank account to make payments and 91.1% of them are active users of social networks. However, it seems that social networks such as Facebook and Twitter are seldomly used to interact or to express a complaint to the provider of financial services.

### 3.3 Dimensions of the digitalization process

**Fig 2:**
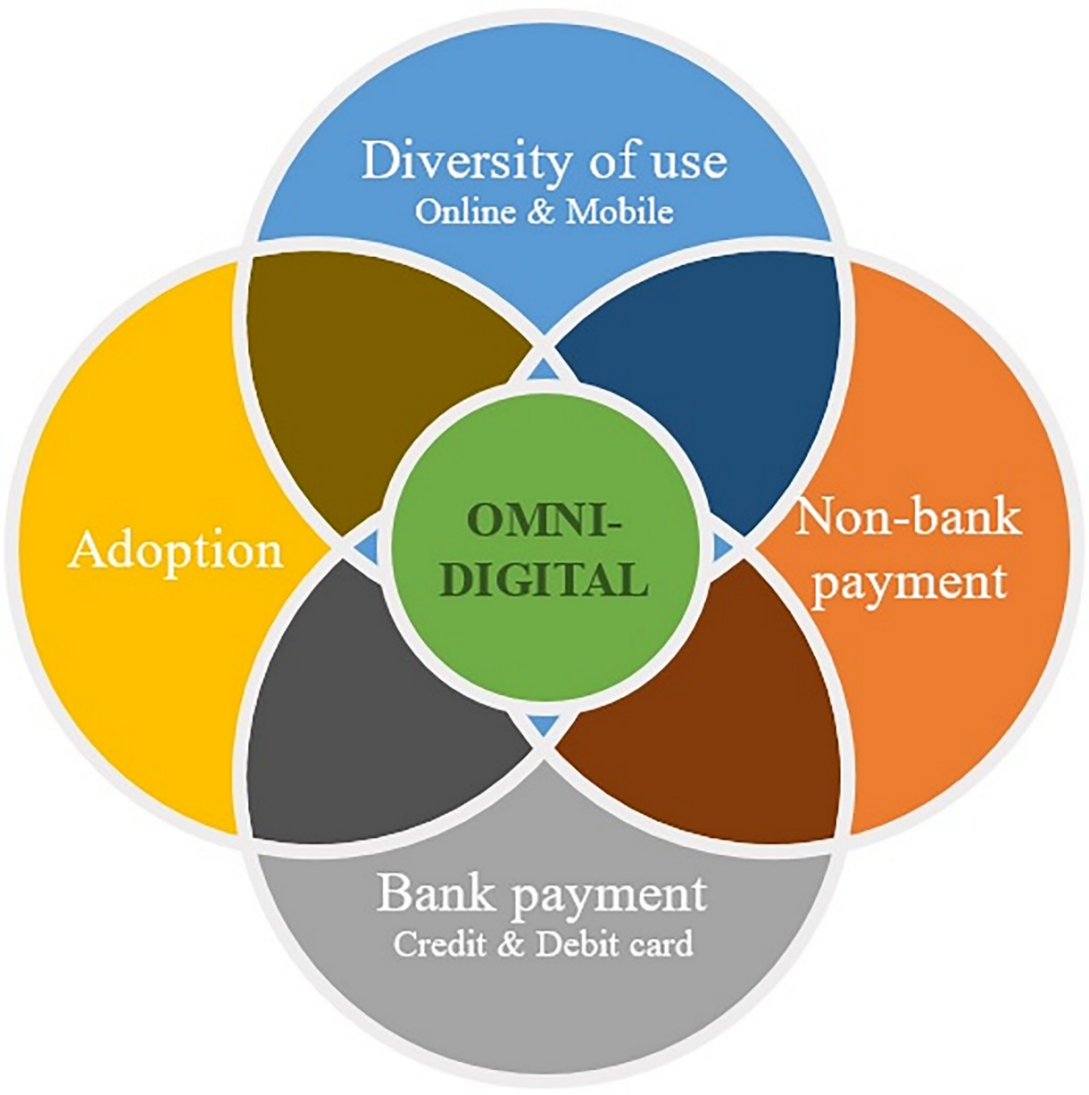
Dimensions of the financial digitalization. Fig 2 plots the dimensions of bank customers’ digitalization: adoption of digital banking, diversification of use, and adoption of bank and non-bank payment instruments.

Going digital is a much broader concept than is commonly understood. Digitalization is not a single dimensional technological expansion but a multifaceted phenomenon. While literature about the global digitalization of societies has examined several dimensions of the digitalization process [55–57], previous studies on the financial digitalization of consumers have mainly focused on the adoption of online channels. As the OECD has suggested, it is convenient to apply a examine a number of dimensions to explore the digital transformation of bank customers. Furthermore, prior findings in the context of online banking—a variety of mechanisms are involved in technology adoption, and the relevance of each one depends on the stage of the adoption [15]—suggest exploring more than one dimension to address issues related to digitalization. Consequently, our study assumes a broad definition of adoption that considers not only the first use of a certain service but also its scope and frequency. Fig 2 plots the main dimensions that we identified from earlier studies (see among others [9, 10, 13, 48, 58–62]): adoption of digital banking, diversification of use, and adoption of bank and non-bank payment instruments. For each dimension, the number of classes is equal to the number of categories in which the individuals are classified (see S3 Appendix in S1 File).

#### • Adoption of Digital Banking

**Fig 3:**
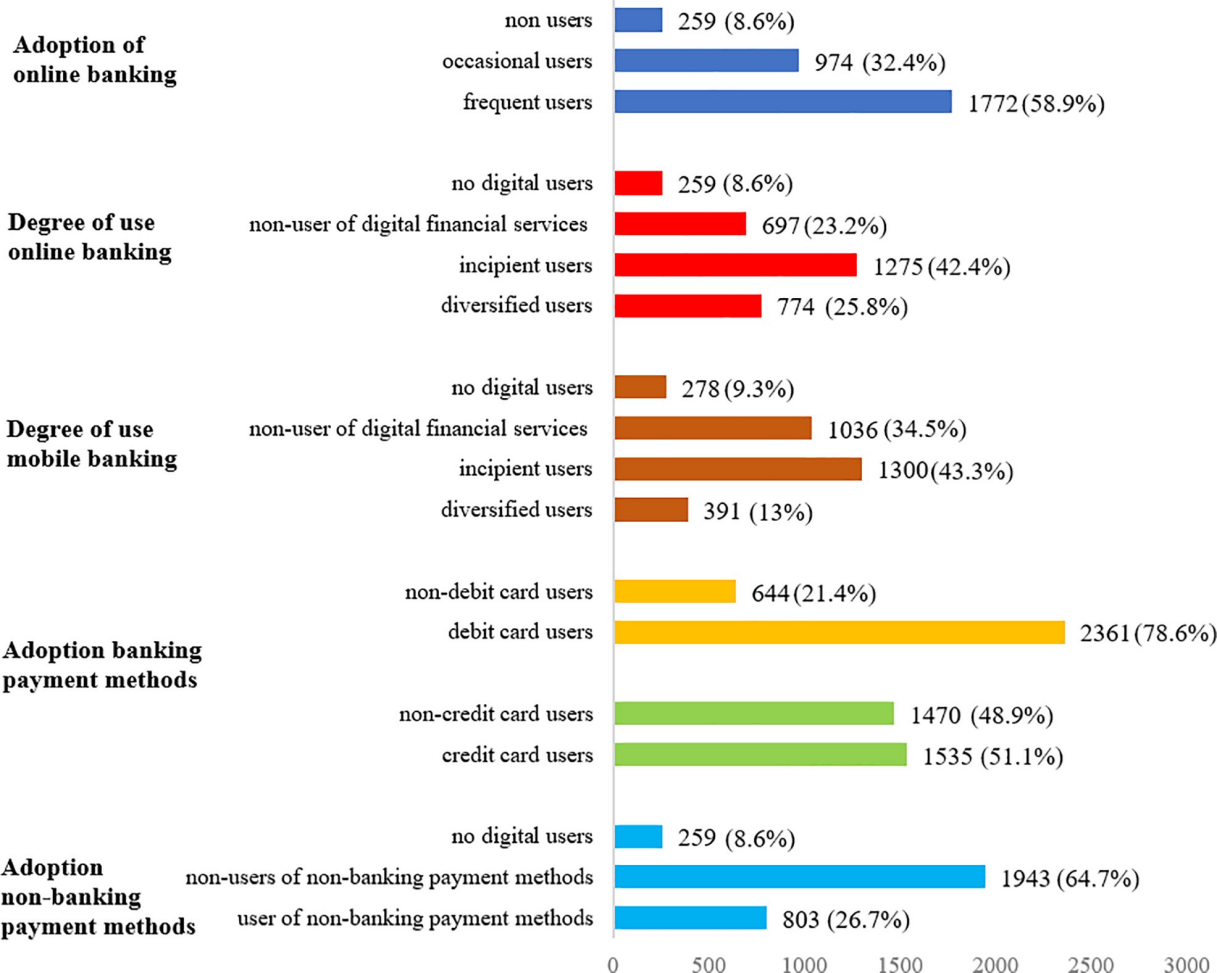
Consumers classification by dimensions (number of surveyed individuals). Fig 3 reports the number of surveyed participants for each dimension considered. The total number of surveyed participants is 3,005.

Regarding the adoption of digital banking we examine 3 classes: non-users, occasional users and incipient users. Non-users are defined as those who over the course of the year have not adopted any kind of financial digitalization, including those who are not even digitalized consumers (i.e., they do not use the internet). Respondents who have become digital customers and conduct online banking activities, but not on a monthly basis, are classified as occasional users. Finally, frequent users are those who conducted online financial activities every month over the course of the year. Fig 3 shows that 59% of the survey participants are frequent users of online financial services, which is consistent with the growth of online banking in Spain officially reported by the European Digital Agenda monitoring exercises.

#### • Diversity of Digital Use

While the initial phase of the digital transformation of consumers involves regular online access, going digital is also related to consumers’ use of diverse digital services. Going digital therefore means conducting a number of financial activities online and not just a single online activity (e.g., just checking one’s account balance).

The factors that drive consumers’ digital diversification might be different depending on the capabilities of the electronic device used to access the service. Therefore, we differentiate between the diversification of online banking users and mobile banking users. As such, survey respondents are classified according to the variety of tasks they carry out (check account balances, pay bills, make transfers, or receive communications). Based on these factors, respondents are then sorted into four categories: no digital users, non-users of digital financial services, incipient users and diversified users.

Individuals who are outside of the digitalization process (i.e., who have no access to the internet) are classified as no digital users. In case of mobile banking, those who do not own a smartphone are also classified as no digital users. Individuals who are frequent internet users but do not conduct any financial activity online are classified as non-users of digital financial services. Incipient users are those who perform some but not all online financial activities at least once a month. Finally, those users that carry out all financial activities online at least once a month are classified as diversified users of digital financial services. Fig 3 reveals that most of the respondents are incipient users, which reflects the worth of exploring this dimension. Bank customers also appear to be customers of digital financial services, but they are still far from being considered “omni-digital” users.

#### • Use of Banks’ Payment Instruments

Although debit and credit cards cannot be considered fully new electronic payment instruments, we also consider them because there has been a technological and safety evolution (i.e. contactless technology). Individuals are divided into 2 classes: non-debit (non-credit) card users and debit (credit) card users. As Fig 3 shows, there is a larger use of debit cards (78%) in comparison to credit cards (51%).

#### • Use of Non-Bank Payment Instruments

While banks have traditionally offered non-cash payment instruments, some technology companies, particularly BigTech and FinTech, have begun to offer non-banking alternatives to pay bills or transfer. Since the adoption of these new means of payment provided by non-financial entities has gained ground, it is interesting to analyze how consumers adopt these alternative means of payments. A non-banking payment instrument is the one that is provided by non-banking institutions (Amazon Pay, PayPal, Google Wallet, Apple Pay, etc.). Regarding the use of these non-bank payment options, 3 classes are considered: non-digital users, non-users of non-bank payment instruments and users of non-bank payment instruments.

Consumers who do not use the internet regularly were classified as non-digital users. Consumers of online financial services who do not use non-bank means of payment were classified as non-users of non-bank payment instruments. Finally, users of non-bank payment instruments include consumers that utilize payment methods of non-bank providers. As illustrated in Fig 3, most respondents are non-users of non-bank payment instruments despite being digitalized.

## 4. Methodology

The human participation in this study was voluntary. It consisted of a telephone survey conducted with all the legal and sociological guarantees by the specialised firm IMOP, as stated in the manuscript. The consent of all the survey participants was informed before conducting the questionnaire. This consent was documented as part of the recorded telephone survey which is guarded by IMOP Insights (C/ Antracita n° 7 Planta 4, 28045 –Madrid). The consent has been verified to be legal according to the Funcas’s Ethics committee and approved by the same Funcas's Ethics Committee, which also ensures that the survey has been conducted according to the principles expressed in the Declaration of Helsinki. Data were analyzed anonymously by the authors.

Most previous studies have employed discrete choice models to examine consumer preferences regarding payments and other financial services [6, 14, 63]. These models, derived from utility theory, are based on maximizing consumers’ utility. Other studies have used structural equations. These structural equations are useful for imputing relationships between latent variables that affect e-banking adoption [12, 15, 43].

However, recent studies have shown that digitalization is a challenging endeavor since there are several and complex factors driving the digitalization of people [20]. Then, this complex patterns suggests that a multidisciplinary approach is required to address digitalization [5]. In doing so, as Delen & Zolbanin [22] argue, machine learning techniques complement to the traditional research methods to address this sort of research questions. Machine learning methods are powerful tools for data mining and permit to take new insights into consumer behavior [21, 23, 24]. In the context of bank customers’ digitalization, machine learning would allow to reveal the complex patterns driving the digitalization process as these algorithms are able to identify complex and nonobvious patterns or knowledge hidden in a database with millions of data points.

In this sense, Bajari, Nekipelov, Ryan, and Yang [64] survey a number of methods used in demand studies to conclude that machine learning techniques are both adequate and effective for this type of analyses as they reveal complex patterns. The advantages of following a machine learning approach in complex contexts, such as consumer behavior, would explain why this machine learning is gaining ground. Miguéis, Camanho, and Borges [65] use a random forest model to find hidden patterns that may be valuable for decision-making in bank marketing. Among others, a machine learning approach is employed to estimate consumer preferences for technology products [26], to examine travel choices [27] or, more generally, to model consumer response [21]. These studies as well as other related research [66–70] indicate that, in a context similar to ours, machine learning algorithms provide greater accuracy (compared to other standard approaches).

Instead of ex-ante selecting a default machine learning technique, we initially consider a number of machine learning techniques that have proved their value in this field (see among others [28–32]. The following machine learning approach is employed to examine bank customer digitalization:

1. Compare a number of machine learning methods arising from many different families and areas of knowledge to select the method that achieves the best performance in terms of accuracy.
2. Employ the selected algorithm to identify the main features driving the bank customer digitalization process.
3. Build a set of classification trees to illustrate the sequence of consumers’ decision-making actions.
4. Use a causal machine learning technique (causal forests) to estimate causal relationships in the digitalization process.

Step 1 and 2 allow us to identify the main features driving the bank customer digitalization process based on the machine learning algorithm with better predictive performance. This way we avoid biases from ex-ante self-selecting a machine learning model. Step 3 allows us to go further in the analysis of bank customers’ digitalization. By estimating a conditional inference tree for each dimension, we may explain the decision-making process. Finally, step 4 allows us to use a causal machine learning algorithm to estimate the impact of the features with the larger predictive power on the digitalization process. Unlike prior studies, this approach allows us to examine what characteristics have a predictive power in explaining the digitalization process (step 1 and 2) but also to explore the decision-making process and the potential effect of these features (causality) on going digital (steps 3 and 4).

All the empirical analyses conducted in the paper are carried out using R software. In each and every case, the models are fed with all the variables extracted from the survey (94 variables—S2 Appendix in S1 File), excluding the outcome. This is the a common procedure in the literature when data comes from a survey specifically designed to examine digital banking (see among others [17, 71–73]). Moreover, as it has been argued in the literature, if the input variables that feed the algorithms are ex-ante filtered or chosen by the researcher, the results obtained would be biased due self-selection process.

Additionally, for those machine learning techniques that require selecting some hyperparameters (e.g. number of features for each tree in the random forest algorithm or C and gamma values in the SVM), they are not arbitrarily chosen but tuned to obtain the optimal parameter values for higher accuracy. The performance of all the machine learning methods and the logit models is computed after having optimized the hyper-parameters for each and every method. In doing so, the following R packages are employed: tune, caret, tuneRF and xgboost.

### 4.1 Machine learning techniques

#### • Random Forest

Random forests are an ensemble of tree predictors in which each tree depends on the values of a random vector sampled independently and with the same distribution for all trees within the forest. Because of the law of large numbers they do not tend to overfit [74]. The algorithm follows these steps:

1. A forest of many trees is grown. Each tree is grown from an independent bootstrap sample derived from the data.
2. For each node of the tree, m variables are independently selected at random out of all M possible variables. Then, on the selected m variables it finds the best split.
3. The algorithm grows each tree to largest extent possible.
4. These steps are iterated over all trees in the ensemble, and the average vote of all the trees is reported as the random forest prediction.

#### • Extreme Gradient Boosting

Gradient boosting is a machine learning technique for regression and classification problems. It came out of the idea of whether a weak learner can be modified to become better. As Valiant [75] argues, the weak learning method is used several times to get a succession of hypotheses, each one refocused on the examples that the previous ones found difficult and misclassified. Then, using a training sample (y, x) the goal of the algorithm is to obtain an estimate of the function F(x) that minimizes the expected value of a loss function over the joint distribution of all the observed values.

Among the gradient boosting methods used in practice, the Extreme Gradient Boosting, is widely used as it is an efficient implementation of the gradient boosting framework. The most important factor behind the success of the extreme gradient boosting is its scalability in all scenarios. Compared to other gradient boosting methods, the extreme gradient boosting use a more regularized model formalization to control over-fitting [76].

#### • K-Nearest Neighbor

The k-nearest neighbors (k-NN) algorithm is a supervised machine learning technique employing a non-parametric method [77]. This algorithm assigns points to the data, compares them using a distance function, and assigns a classification based on the labels of the nearest points. The data point which is located at the minimum distance from the test point is assumed to belong to the same class. One of the advantages of this algorithm is that it does not derive any discriminative function from the training data. That is why the k-nearest neighbor algorithm is called a lazy learner or Instance based learning. Moreover, the k-NN algorithm is robust to data that contains a lot of noise and it is able to handle data with multiple classes.

In line with prior studies, the most common distance functions used in the literature are employed: the Euclidean distance $\sqrt{\sum_{i=1}^{k}{\left(x_{i}-y_{i}\right)}^{2}}$, the Manhattan distance $\sum_{i=1}^{k}\left|x_{i}-y_{i}\right|$ and the Chebyshev distance $\underset{i}{\max}\left|x_{i}-y_{i}\right|$. The number of neighbors considered and the weighted functions (kernel) are tuned in order to optimize to maximize the performance. All the 3 distance functions—Euclidean, the Manhattan distance and the Chebyshev—are used to compute the K-nearest neighbor algorithm.

#### • Support Vector Machine

A support vector machine (SVM) is a supervised machine learning model that uses classification algorithms to solve a prediction problem for a discrete outcome using a vector of regressors, initially developed by Vapnik [78]. The algorithm constructs an optimal hyperplane that correctly classifies data points by separating the points of categories as much as possible [79]. The closest values to the classification margin are known as support vectors while the goal is to maximize the margin between the hyperplane and the support vectors [80].

Empirically, the kernel used in training the support vector machine includes the linear, radial, polynomial and sigmoid functions.

#### • Bayesian Networks

A Bayesian network is a direct acyclic graph encoding assumptions of conditional independence. In a Bayesian network, nodes are stochastic variables and arcs are dependency between nodes. A Bayesian network is defined by the nodes, a finite set N = {A,B,…} of nodes (vertices), arcs, a set L of arcs (edges) and a joint probability density function. Then, for any set of random variables (X), the probability of any member of a joint distribution can be calculated from conditional probabilities as follows:

$$P(X_{1}=x_{1},\ldots ,X_{n}=x_{n})=\prod_{v=1}^{n}P(X_{V}=x_{v}|X_{v+1}=x_{v+1},\ldots ,+X_{n}=x_{n})$$

Bayesian network classifiers [81, 82] are competitive performance classifiers [83]. In this sense, a Bayesian network classifier is simply a Bayesian network applied to classification. Specifically, the prediction of the probability of some discrete (class) variable Y given some features X. Together with the well-known Naive Bayes classifier [84] more elaborate models exist taking advantage of the Bayesian network [85, 86] such as the averaged one-dependence estimators (AODE) [87], the Chow-Liu’s algorithm for one-dependence estimators (CL-ODE) [81], the forward sequential selection and joining (FSSJ) [88], the backward sequential elimination and joining (BSEJ) [88], the Hill-climbing tree augmented naive Bayes (TAN-HC) [89] and the Hill-climbing super-parent tree augmented naive Bayes (TAN-HCSP) [89].

## • Artificial Neural Networks: Extreme learning machine

Extreme learning machine (ELM) is a type of artificial neural network, called feedforward neural networks, which randomly chooses hidden nodes and analytically determines the output weights of single-hidden layer feed forward neural networks (SLFNs). The learning speed is thousands of times faster than traditional feedforward network learning algorithms like the back-propagation (BP) algorithm [90]. Moreover, compared with the conventional neural network learning algorithm it overcomes the over-fitting problem [91]. Mathematically, given a training set, an activation function and a hidden node, the algorithm follows three main steps:

1. It assigns randomly input weight *w_i_* and bias *b_i_*, (i = 1,… N).
2. It calculates the hidden layer output matrix
3. It calculates the output weight

The activation function commonly used include the sigmoidal functions as well as the radial basis, sine, hard-limit, symmetric hard-limit, satlins, tan-sigmoid, triangular basis, rectifier linear unit and linear function.

### 4.2 Logit Model

Since prior literature has mainly employed discrete choice models to examine customers’ behavior, we also employ logit models to examine bank customer digitalization where *Y*{\*displaystyle y*^{*}^}*YY* is the level of bank digitalization for each dimension of financial digitalization considered, *X* = (*x*_1_,…,*x_n_*) is the set of variables and *i* = (1,… j) are the different categories for each dimension.

We employ an ordered logit regression for the adoption decision and the diversification of digital usage and a simple conditional logit—for the adoption of bank or non-bank payment instruments. To be consistent, the same set of variables used in the machine learning methods are employed.

### 4.3 Conditional inference trees

We use the characteristics and determinants with the largest discriminant power to build a decision tree for each dimension by estimating a conditional inference tree. This technique estimates a regression relationship by binary recursive partitioning in a conditional inference framework. In order to build the trees for each dimension, we follow the methodology developed by Hothorn, Hornik, & Zeileis [92] and Hothorn, Hornik, Van DeWiel, et al. [93]. The algorithm tests the global null hypothesis of independence between each of the input variables and the response and selects the input variable with the strongest association to the response. The algorithm then implements a binary split in the selected input variable and recursively repeated this process for the each of the remaining variables. The classification tree infers the sequencing of customers’ decision-making process, which helps to explain how bank customers go digital. This is particularly relevant since those classification trees do not require any linearity assumptions, which is important because many of the digitalization determinants could be nonlinearly related.

### 4.4 Causal machine learning

Since machine learning models are not designed to estimate causal effects, a new field of study has emerged very recently, the causal machine learning. Over the last few years, different causal machine learning algorithms have been developed, combining the advances from machine learning with the theory of causal inference [94]. The aim of these causal machine learning techniques is to complement the machine learning methods by estimating causal effects, rather than to substitute them [95–97]. The main advantage of causal machine learning is that it can be used after the modeling phase in order to confirm some of the relations between variables and the target/outcome. In our context, by employing a causal learning method we aim to examine the causal effect of those features with the larger predictive power on the digitalization process.

Among the recent methods developed in the causal machine learning literature, causal forest have gained relevance [95–97]. Knaus et al. [98] show that causal forests perform consistently well across different data generation processes and aggregation levels. The causal forest algorithm [96] is a forest-based method for treatment effect estimation that allows for a tractable asymptotic theory and valid statistical inference extending Breiman’s random forest algorithm.

Methodologically, causal forests maintain the main structure of random forests—including recursive partitioning, subsampling, and random split selection- but instead of averaging over the trees they allow to estimate heterogeneous treatment effects (causality) [99]. Then, compared to a regular decision tree, the causal tree uses a splitting rule that explicitly balances two objectives: first, finding the splits where treatment effects differ most, and second, estimating the treatment effects most accurately. In order to obtain consistent estimates of the treatment effects (the features that may have an impact on digitalization) it splits the training data into two subsamples: a splitting subsample and an estimating subsample [97, 99]. The splitting subsample is used to perform the splits and thus grow the tree and the estimating subsample is then used to make the predictions. All observations in the estimating subsample are dropped down the previously grown tree until it falls into a terminal node. So, the prediction of the treatment effects is then given by the difference in the average outcomes between the treated and the untreated observations of the estimating subsample in the terminal nodes. Athey & Wager [99] provide a full mathematical explanation on how causal forests are built for causal inference.

Using this novel empirical methodology, we are able to examine the causal effect of those features with the larger predictive power on the digitalization process. Then, the level of digitalization is not our main interest but the impact of those features with the larger predictive power on the digitalization process. All analyses are carried out using the R package grf [100]. To run this causal algorithm, we take a conservative approach assuming that the level of digitalization of the customers can be arbitrarily correlated within a bank. Sample individuals are customers of 33 different banks. Hence, the errors are clustered at the bank-level, and we have a total of 33 clusters/banks.

## 5. Results

### 5.1 Model selection

In order to select the model with the best performance, being consistent with the standard practice followed in the machine learning literature, we randomly selected 70% of the data as training data (2,104 observations) and designated the remaining data (901 observations) as test data. By doing so, we are able to determine the accuracy of the model ensuring that the algorithm is actually finding real patterns in the data and not just overfitting.

The performance of the models is compared by computing several metrics. Consistent with earlier machine learning studies (see among others [101–105]), we use accuracy as a measure of performance. It is defined as the number of correctly predicted data points out of all the data points. Moreover, we also compute additional standards metrics: precision (the number of correctly identified positive results divided by the number of all positive results, including those not identified correctly), recall (the number of correctly identified positive results divided by the number of all individuals that should have been identified as positive) and F1 score (the harmonic mean of the precision and recall). While recall tells us about the sensitivity of the model and precision provides information about its positive predictive value, the advantage of the F1 score is that it combines both metrics. A high F1 score is a sign of a well-performing model, even in situations where you might have highly imbalanced classes. Finally, for those dimensions with three or more classes (multi-classes) we also compute the Macro F1 score which is the averaged F1.

For the sake of brevity, Table 2 reports just the results for the best model identified per machine learning method after having optimized the hyper-parameters for each and every method. The forecasting accuracy for those cases in which several models (using several kernels and activation functions) are estimated could be found in S4 Appendix in S1 File reports. Moreover, to save space in Table 2, we just report the precision, recall and F1 score for the class which is more frequent among the survey participants (see Fig 3). Overall, Table 2 shows that the random forest algorithm provides the highest level of accuracy for all the dimensions considered. Random forests accurately predict 88.41% of bank customers’ online banking adoption profile, 70.11% of the diversity of digital use of online banking, 70.01% of the diversity of digital use of mobile banking, 85% (74.89%) of debit (credit) card adoption, and 76.14% of non-bank payment instruments adoption. The second best method is the extreme gradient boosting algorithm, which also present a high percentage of accuracy.

**Table 2:** Models’ performance.

		Random forest	Extreme Gradient Boosting	K-Nearest Neighbor	Supportive Vector Machine (SVM)	Bayesian Networks	Artificial Neural Networks: Extreme learning machine	Logit
				Euclidean	Radial	FSSJ	Rectifier Linear Unit	
**Adoption of online banking**	*Accuracy*	88.41%	84.99%	84.92%	84.58%	86.48%	82.18%	79.27%
	*Precision*	94.01%	91.99%	84.66%	90.21%	90.71%	88.74%	54.63%
	*Recall*	88.79%	85.56%	90.19%	85.07%	87.93%	81.83%	43.28%
	*F1 score*	91.33%	88.66%	87.34%	87.56%	89.30%	85.15%	48.30%
	*Macro F1 score*	91.41%	85.75%	88.15%	87.09%	88.62%	83.28%	72.05%
**Diversity of digital use: online banking**	*Accuracy*	70.11%	68.82%	63.41%	67.36%	66.36%	65.41%	55.01%
	*Precision*	67.76%	72.41%	58.96%	70.94%	63.39%	65.54%	55.10%
	*Recall*	71.56%	62.19%	70.81%	63.70%	60.73%	61.56%	64.00%
	*F1 score*	69.61%	66.91%	64.35%	67.12%	62.03%	63.49%	59.22%
	*Macro F1 score*	74.82%	74.24%	67.54%	72.90%	73.25%	71.53%	53.42%
**Diversity of digital use: mobile banking**	*Accuracy*	70.01%	67.85%	63.97%	66.27%	63.98%	61.75%	59.57%
	*Precision*	68.52%	71.31%	57.93%	68.10%	68.00%	68.52%	62.44%
	*Recall*	75.08%	61.19%	75.37%	60.62%	59.86%	60.09%	64.08%
	*F1 score*	71.65%	65.86%	65.51%	64.14%	63.67%	64.03%	63.25%
	*Macro F1 score*	69.51%	66.89%	63.80%	67.57%	68.65%	69.29%	56.85%
**Debit card**	*Accuracy*	85.00%	84.79%	80.60%	82.11%	84.43%	83.26%	84.23%
	*Precision*	92.47%	92.63%	88.23%	95.51%	92.94%	92.32%	95.76%
	*Recall*	89.73%	86.96%	89.34%	85.34%	88.24%	84.64%	85.84%
	*F1 score*	91.08%	89.70%	88.78%	90.14%	90.53%	88.31%	90.53%
**Credit card**	*Accuracy*	74.89%	73.51%	64.75%	72.63%	72.16%	74.84%	70.62%
	*Precision*	74.68%	77.51%	65.57%	74.06%	77.60%	74.13%	69.26%
	*Recall*	76.87%	70.44%	71.97%	73.75%	70.84%	76.47%	76.58%
	*F1 score*	75.76%	73.81%	68.62%	73.90%	74.06%	75.28%	72.74%
**Adoption of Non-bank payment methods**	*Accuracy*	76.14%	75.91%	74.94%	74.48%	70.91%	75.84%	73.46%
	*Precision*	82.02%	86.92%	76.97%	86.61%	84.11%	88.97%	78.43%
	*Recall*	85.17%	77.81%	87.29%	77.98%	74.92%	77.01%	82.08%
	*F1 score*	83.56%	82.11%	81.80%	82.07%	79.25%	82.56%	80.21%
	*Macro F1 score*	73.51%	76.62%	73.58%	72.88%	71.59%	76.02%	72.05%

Table 2 reports the performance measures for all the models (machine learning algorithm and logit) employed in examining the digitalization of bank customers. Precision, recall and F1 score are obtained for the class which is more frequent among the survey participants.

Regarding the F1 and macro F1 scores, the random forest model also seems to provide the highest values. For example, the macro F1 score is 91.41% for the adoption of online banking. The higher F1 and macro F1 scores of the random forest, together with the highest predicted accuracy, suggest that the random forest is the machine learning method that exhibits the best performance. Furthermore, we also observe that the performance obtained using most of the machine learning techniques, but specially the random forest, outperforms the standard logit and ordered logit models.

The higher predicted accuracy of the random forest algorithm is in line with prior studies. Bajari, Nekipelov, Ryan, & Yang [64] compare several methods and based on the out-of-sample prediction error shows that the random forest is the most accurate. Similarly, Fernández-Delgado et al. [102] evaluate 179 machine learning algorithms arising from 17 families to conclude that random forests provide the best results in terms of predicted accuracy. Consequently, since in our case the random forest is the most accurate algorithm, this algorithm is employed in order to identify the main features driving the bank customers’ digitalization process.

### 5.2 Validity

Finally, in order to check the stability of the accuracy of the results, we employ two cross validation methods: the k-fold cross-validation and the repeated K-fold cross-validation. In doing so, the dataset is split into 10 groups (k = 10), since this value has been shown empirically to yield test error rate estimates that suffer neither from excessively high bias nor from very high variance [106, 107]. In case of the repeated K-fold cross-validation, the data is split into 10-folds, repeating the process five times. The results reported in Table 3 confirm the validity of the models employed.

**Table 3:** Random forest hyperparameters and cross-validation of the algorithm.

	**Panel A. Random Forest Hyperparameters**					
	Adoption of online banking	Diversity of digital use: online banking	Diversity of digital use: mobile banking	Debit card	Credit card	Adoption of Non-bank payment methods
**Number of Trees**	1,000	1,000	1,000	1,000	1,000	1,000
**Number of Features for each Tree**	13	22	22	15	16	13
**Maximum Depth of the Tree**	20	20	20	20	20	20
	**Panel B. Cross-validation accuracy**					
**K-fold cross validation**	86.62%	69.19%	68.85%	75.44%	74.79%	76.17%
**Repeated K-fold cross-validation**	86.74%	68.94%	69.32%	75.57%	74.93%	76.28%

Panel A of Table 3 reports the values of the main hyperparameters to estimate the random forest model: number of trees, number of features for each tree and the maximum depth of the tree. Panel B of Table 3 reports the accuracy of the random forest algorithm by two cross-validation exercises: k-fold cross validation (k = 10) and repeated K-fold cross-validation (k = 10, repetitions = 5). These values are reported for all the dimensions of digitalization examined.

For replicability purposes -and given that the random forest is the selected algorithm- Table 3 reports the optimal hyperparameters of this algorithm for each dimension of financial digitalization. The Out-of-bag (OOB) error remains stable if more than a thousand trees are built. Then, since the improvement is mostly insignificant, the number of trees is set to 1,000. Moreover, since taller trees allow the model to learn very specific relationships between the features splitting the nodes and our data set [108], it is important to limit the depth of the tree in order to avoid overfitting. In doing so, we allow up to 20 nodes from the root down to the furthest leaf node.

### 5.3 Features of the digitalization of bank customers

**Fig 4:**
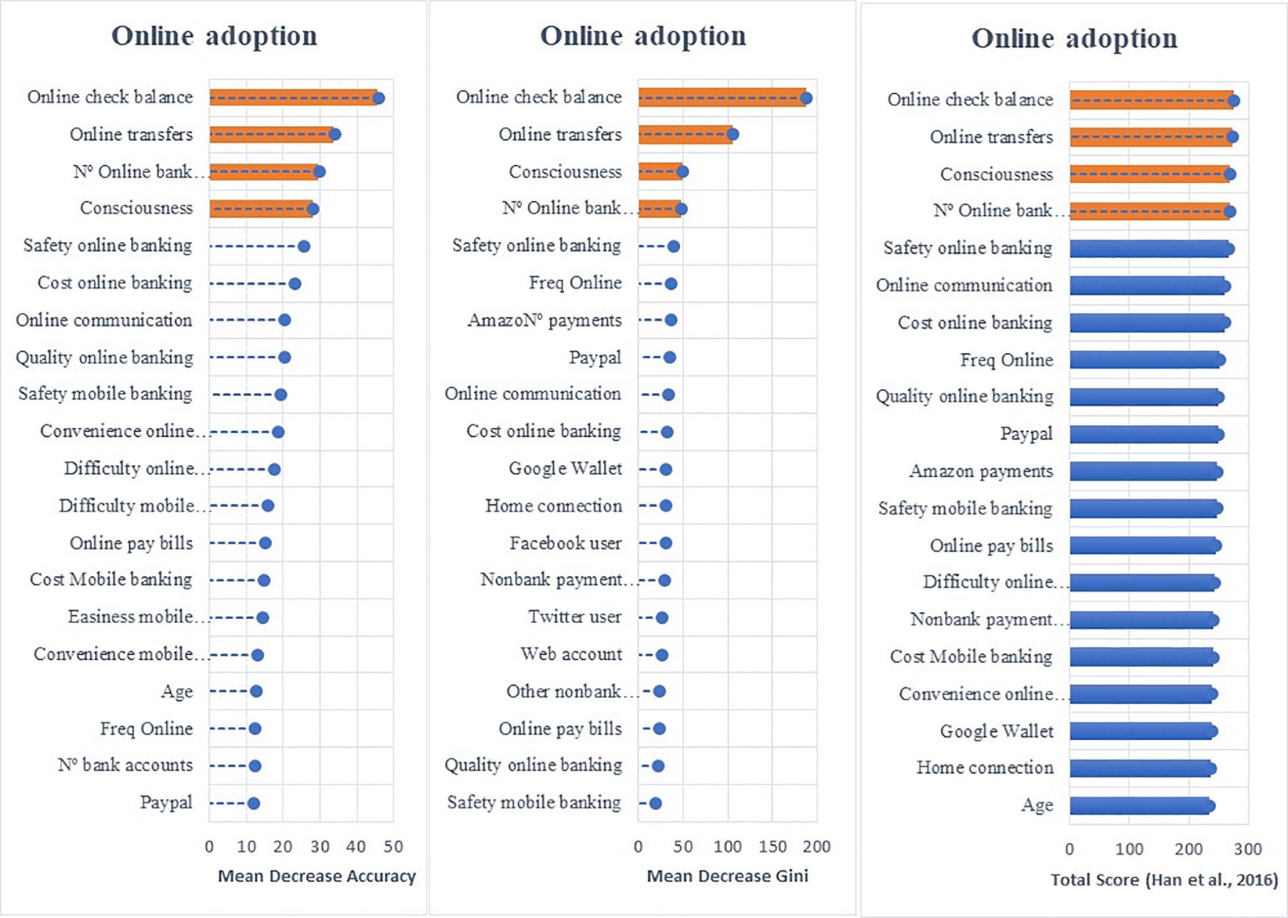
Variable importance for the random forest model on online banking adoption.

**Fig 5:**
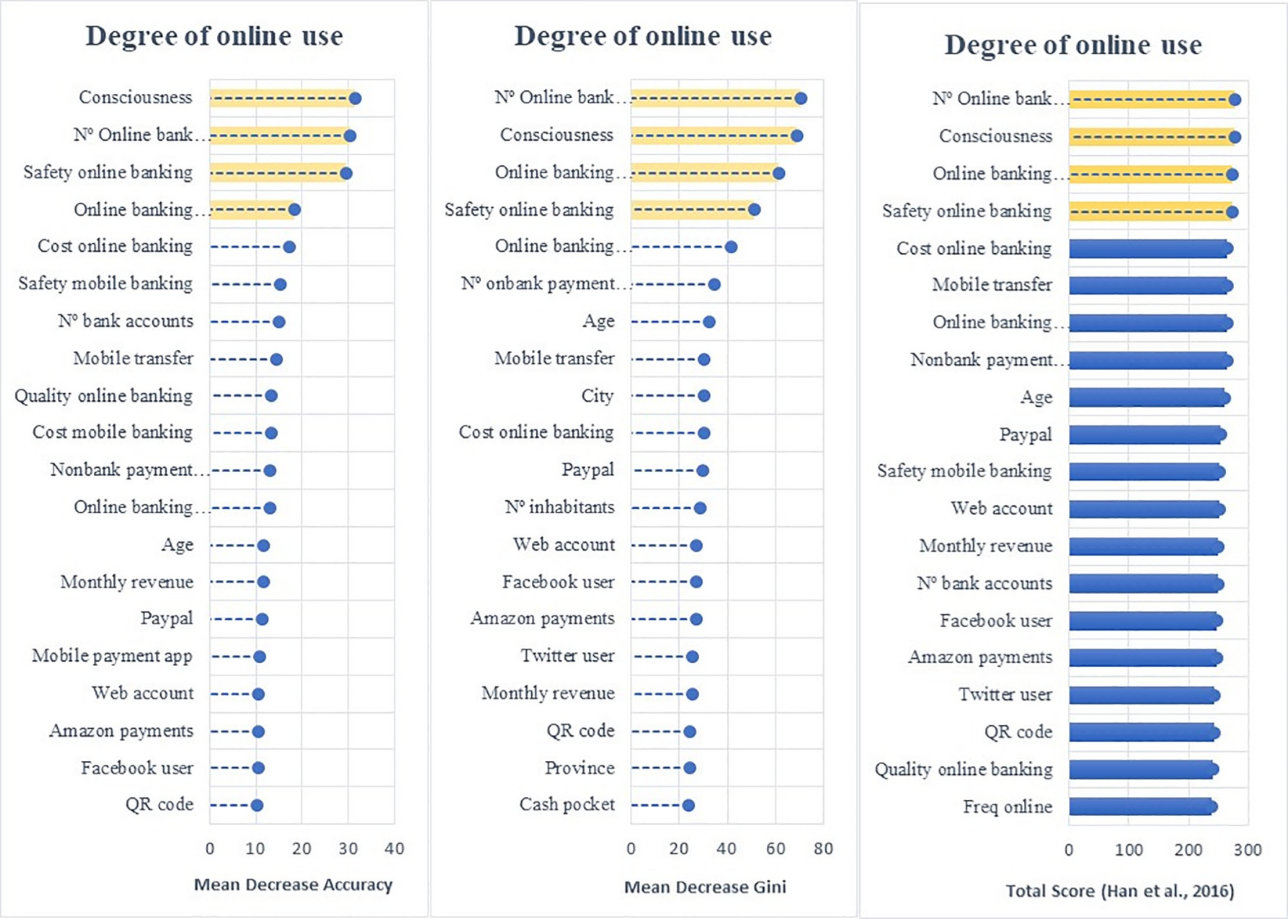
Variable importance for the random forest model on diversification of online banking uses.

**Fig 6:**
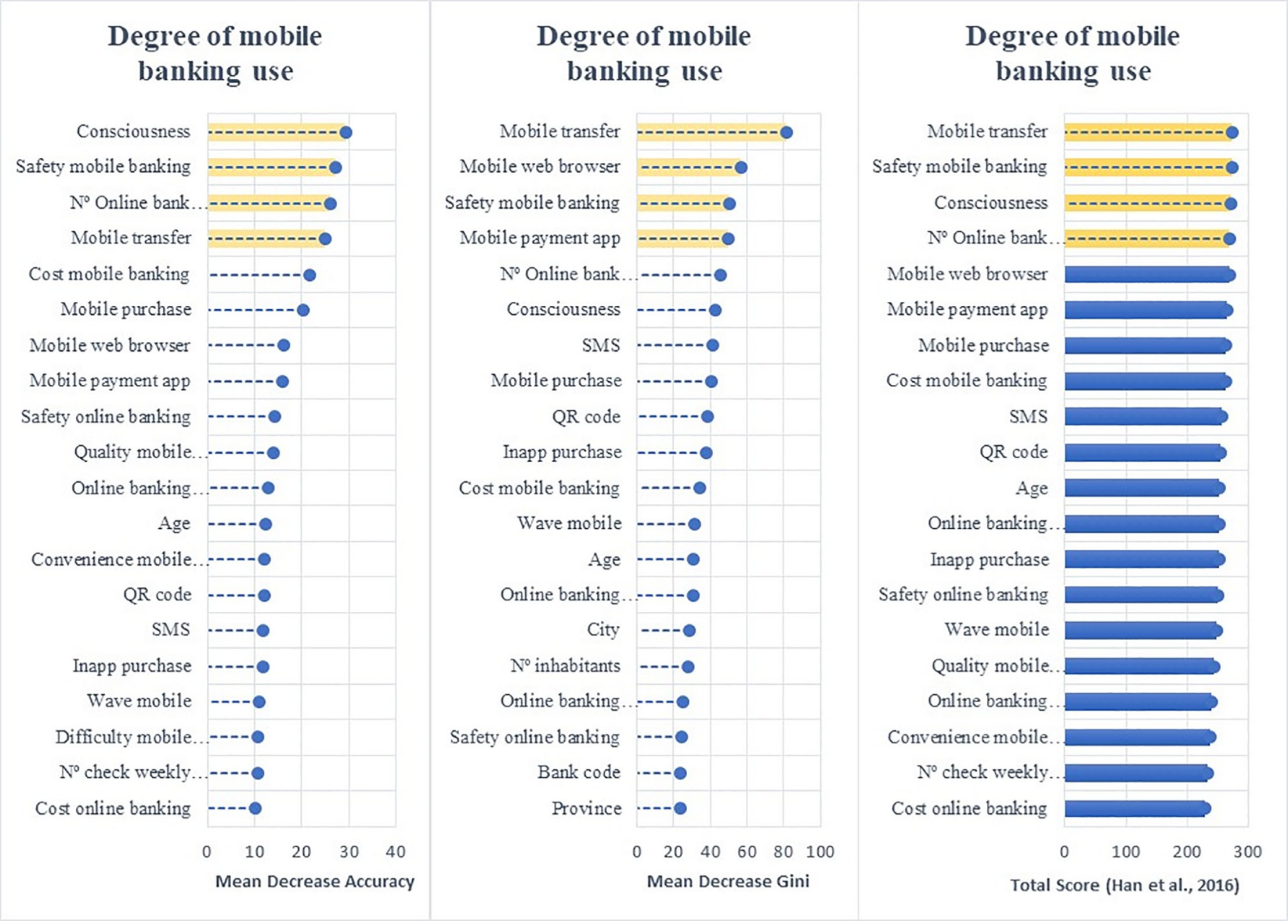
Variable importance for the random forest model on diversification of mobile banking uses.

**Fig 7:**
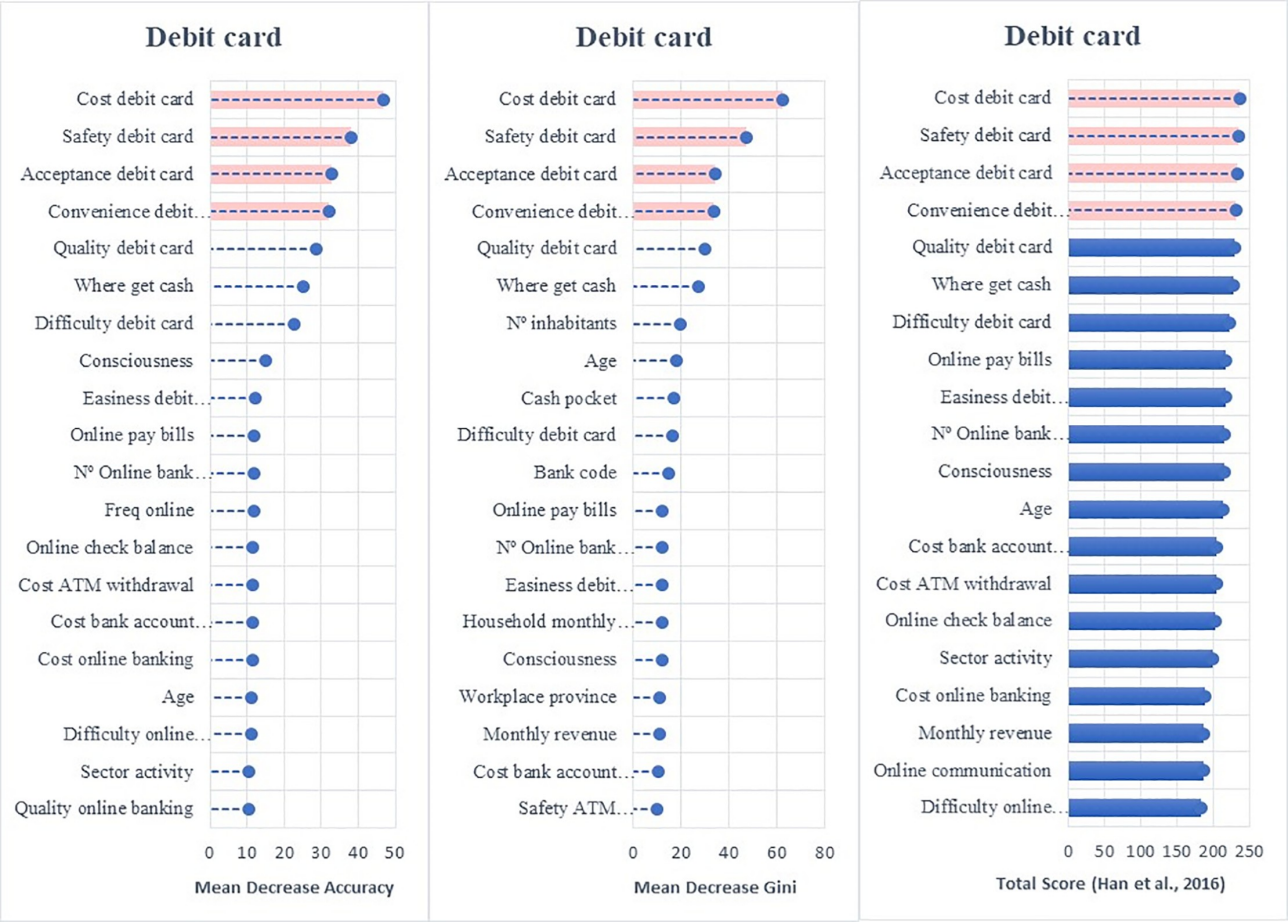
Variable importance for the random forest model on debit card adoption.

**Fig 8:**
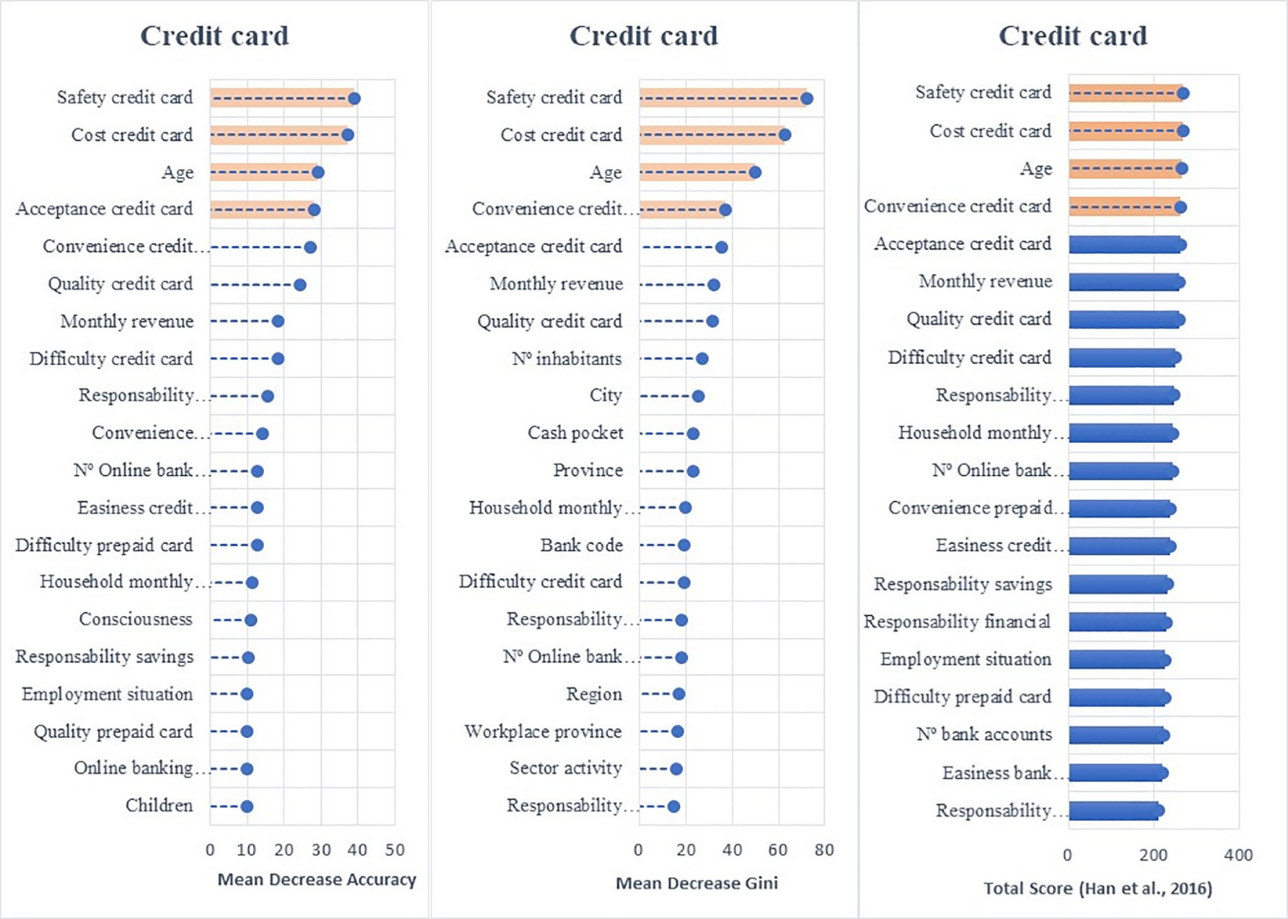
Variable importance for the random forest model on credit card adoption.

**Fig 9:**
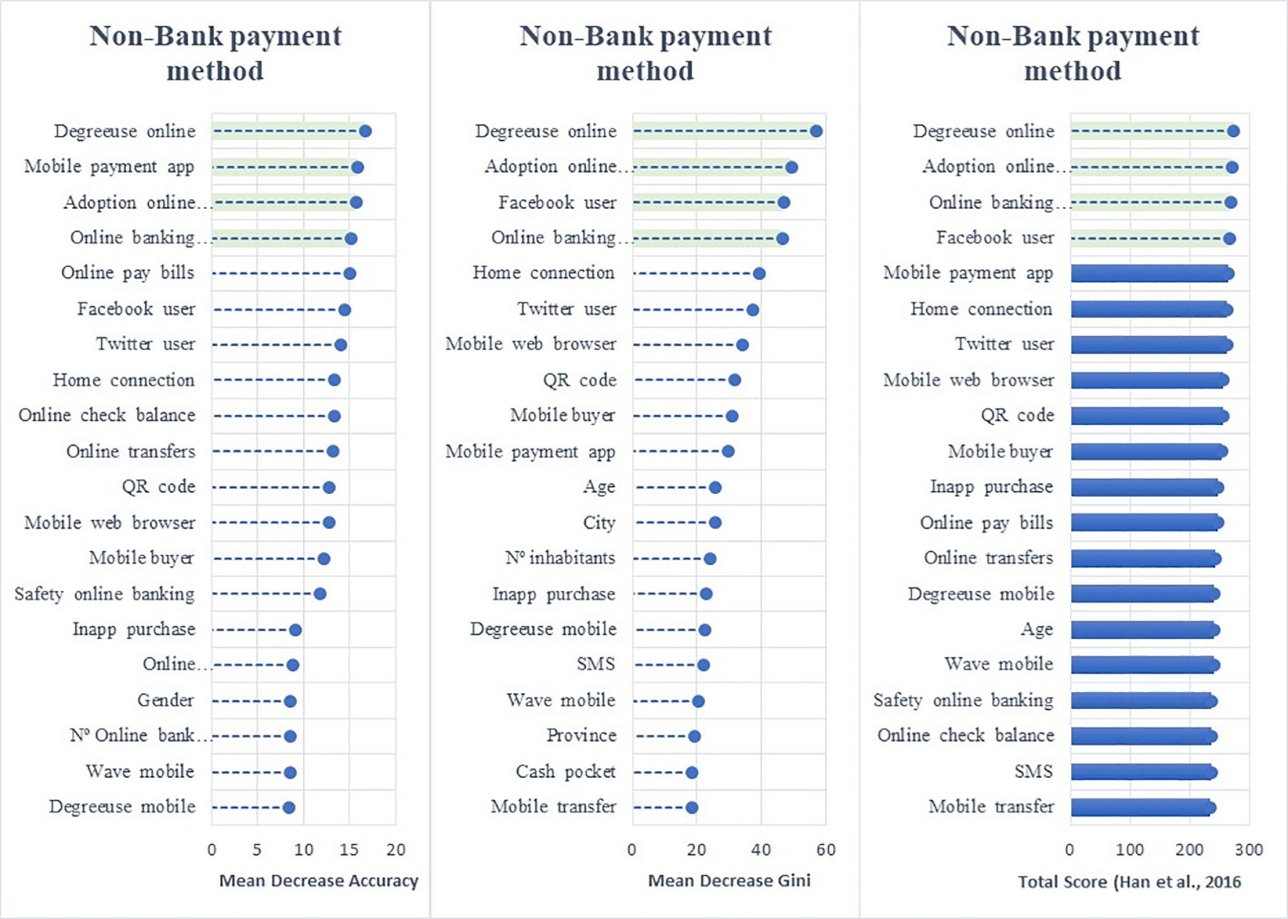
Variable importance for the random forest model on adoption of non-bank payment methods. Figs 4 to 9 report the plots showing the relative statistical importance of each feature in the classification. The left-hand side graph shows the Top 20 features by Mean Decrease in Accuracy. The centered graph shows the Top 20 features by Mean Decrease in Gini. The righ-hand side graph shows the Top 20 features by Total Score [109].

Employing the random forest algorithm [74] we identify the features with the largest power in predicting bank customers’ digitalization reporting the relative statistical importance of each factor in the classification of individuals by their digital profiles (Figs 4 to 9). The determinants and characteristics are plotted on the *y*-axis ranked by their absolute level of importance while their relative importance is charted on the *x*-axis. The mean decrease in accuracy reflects the mean loss in accuracy when each specific variable is excluded from the regression algorithm. Therefore, the determinants and characteristics with the greater mean decrease in accuracy are the most relevant for the classification of bank customers. Additionally, the mean decrease in Gini is a measure of how each feature contributes to the homogeneity between the decision trees used in the resulting random forest. Furthermore, besides reporting the mean decrease in accuracy and the mean decrease in Gini for each variable, we employ the variable selection procedure MDAMDG proposed by Han et al. [109]. It consists of 1) running the random forest algorithm and returns the mean decrease in accuracy and the mean decrease in Gini of each variable 2) ranking every variable using the mean decrease in accuracy and the mean decrease in Gini, respectively, 3) scoring each variable 4) computing the total score of each variable 5) reordering them by the total score. While, as abovementioned, all the variables extracted for the survey (S2 Appendix in S1 File) are used to feed the algorithm as input features, for the sake of brevity Figs 4 to 9 report only the top 20 features by their relative importance. These Figures provide the rank of the variables based on the mean decrease in accuracy, mean decrease in Gini and the total score [109].

#### • Adoption of Digital Banking

The machine learning algorithm reveals that the first-order factors determining the adoption of digital banking are *online check balance* (whether account balances are checked online), *number of online bank accounts*, *online transfers* (whether the customer has made an online bank transfer in the last three months) *and consciousness* (degree to whether the customer is conscious that an online access is available).

These results suggest that the relevant factors in going digital are those related to customers becoming accustomed to the online channels by checking their bank account balances or transferring money and being aware that these activities can be conducted online. Bank customers’ perceptions of security, cost, and ease of use of banking services were found to be secondary factors in going digital. As in other industries, consumers tend to go through several stages of adoption: awareness, consideration, and choice. Our results confirm the significance of awareness in the multistage process of going digital.

#### • Diversity of Digital Use: Online and Mobile Banking

Figs 5 and 6 show the baseline random forest results in terms of the diversification of online and mobile banking services, respectively. The *number of online bank accounts*, *consciousness* (being aware of the possibility of having access to online services), *safety of online banking* (how customers perceive the level of security of online banking) and *online banking communication* (whether customers have used online services or e-mail as their communication method with their bank) are the features with the largest influence on diversifying the use of online banking.

Considering both the adoption and diversification of digital use, we argue that the digitalization process originates from the customers’ need to check their bank account balances and transfer money. However, being aware of the possibility of accessing financial services through online banking and the perceived safety of operating online are the main factors to diversify the use of online banking services. Furthermore, the digitalization of the communication channel between customers and banks also fosters the diversification of customers’ online activities.

Regarding the diversification of the use of mobile banking, we find that the factors with the greatest predictive power are the *number of online bank accounts*, *safety mobile banking*, *consciousness and transferring money via mobile*.

Overall, the algorithm reveals that online and mobile diversification are driven by common features: consciousness of the possibilities offered by digital banking, the perceived level of security of the channel used, and the number of digital bank accounts available. However, it is worth noting that transferring money was a distinct factor in determining the diversification of mobile banking. It seems that money transferring via mobile may become the gateway to other digital financial activities. This finding partially explains the importance of the irruption of FinTech companies in the payment sector compared to other financial services.

#### • Use of Banks’ Payment Instruments: Debit and Credit Cards

The main factors that influence the use of debit and credit cards (see Figs 7 and 8) are the perceived *cost*, *safety*, *acceptance and convenience* of these payment instruments. Unlike the adoption and penetration of online and mobile banking, the use of debit and credit cards seems to be dominated by bank customers’ perceptions of cards’ cost, safety, and acceptance. It is interesting to see that merchants’ acceptance of debit and credit cards as payment instruments is relevant since it determines their utility, which could explain why bank customers are concerned about ensuring their acceptance before adopting them as regular payment instruments. This result suggests that the technological changes linked to cards (CVC code, EMV chips, contactless technology, multi-factor authentication) have been evolving and affect customers’ perceptions of safety and convenience.

#### • Use of Non-Bank Payment Instruments

Fig 9 illustrates that the adoption of non-bank payment methods is driven by *mobile payment app* (whether customers’ use of mobile apps to make payments), *frequency and degree of online banking*, *online banking complaint (*whether customers’ use online channels to lodge a complaint with the bank) and being active on social media (*Twitter and/or Facebook user)*. These findings reveal that the prior profile as digital bank customer (frequency and scope using online banking) as well as being already using payment apps determine the use of alternative payment methods. Moreover, the relevance of using online channel to complain may reveal that a certain level of dissatisfaction with the bank may lead bank customers to adopt non-bank means of payment.

Overall, while prior theories and studies have given prominence to the technological components of the service and to consumers’ perceptions to explain the digital jump (see among others [10, 12, 13, 34, 110]), our approach reveals that customers go digital first for information-based needs and, later, to undertake transactional services. Customers’ perceptions also play a role but only to explain the scope of the digitalization (being a diversified digital customer). However, customers’ perceptions (in particular, safety and cost), are particularly related to the use of bank payment methods (credit and debit cards). Moreover, the adoption of non-bank payments seems to be driven by the prior adoption and usage of online and mobile banking services.

#### • Robustness and stability over subsamples

Finally, for robustness purposes we also employ the second best algorithm in terms of accuracy (Table 2), the extreme gradient boosting, to identify the features with the largest predictive power. The figures in S5 Appendix in S1 File plot the most important features that predict bank customer digitalization based on this algorithm. The relative importance of each feature is computed using the contribution of the corresponding feature for each tree in the model (Gain). Overall, they show that the features with the largest predictive power according to the random forest algorithm are also identified as the most important by the extreme gradient boosting algorithm. Since both methods coincide on the main customers’ features predicting the level of digitalization, this adds robustness to the ability of machine learning methods to reveal the characteristics that drive customers’ digitalization.

Furthermore, we also aim to ensure that when feeding different data to the algorithm the predicted accuracy was stable. In doing so, we employ different subsamples based on socio-economics characteristics—gender, age, and habitat—to go through the machine learning process in order to show the robustness in terms of accuracy. Young people are those between 18 and 34, while old people are over 55 years old. The rural areas category includes people living in municipalities with less than 10,000 inhabitants while the urban category includes those living in cities with more than 200,000 inhabitants.

**Fig 10:**
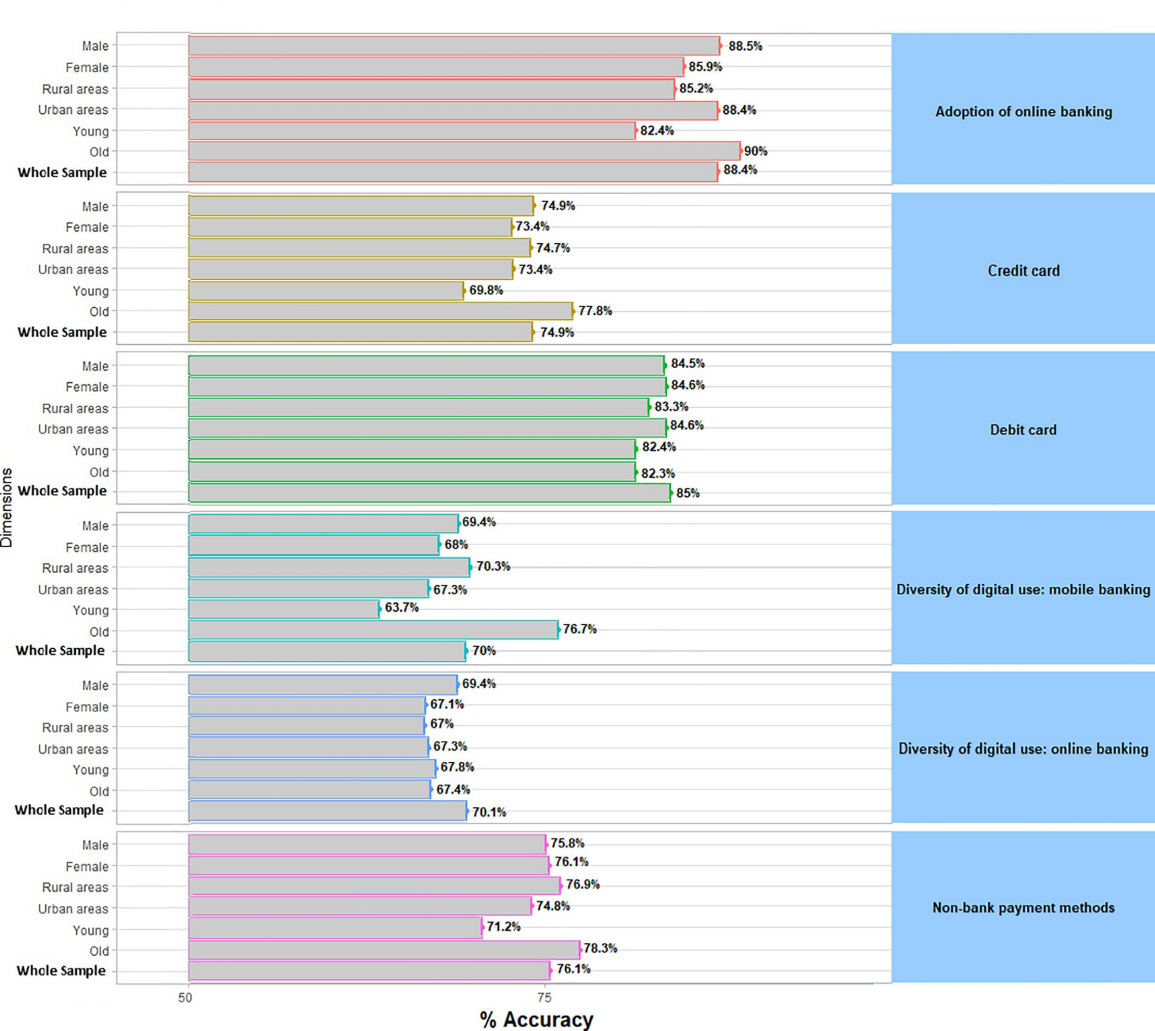
Stability over subsamples. Fig 10 plots the predicted accuracy of the random forest algorithm across the different subsamples: based on gender (male vs female), age (young vs old) and habitat (rural vs urban areas).

Fig 10 shows the accuracy across the different subsamples- based on gender, age, and habitat—that feed the model. As it could be observed the performance of the algorithm across these three subsamples remains similar to the whole performance when the algorithm is fed with the entire dataset. This result shows that the performance of the algorithm when examining the digitalization of bank customers is stable, which means, that it is not dependent on the sample subset used to feed the model. This is relevant since it reveals that the machine learning algorithm does not overfit bank customers’ digitalization for a particular profile of customers.

### 5.4 Bank customers’ digitization trees

The characteristics and determinants with the largest discriminant power are employed to estimate a conditional inference tree for each dimension. This technique estimates a regression relationship by binary recursive partitioning in a conditional inference framework. As already mentioned, these trees are built following the methodology developed by Hothorn, Hornik, & Zeileis [92] and Hothorn, Hornik, Van DeWiel, et al. [93]. In doing so, those variables with the largest relative importance based on Han et al. [109]’s total score, which accounts for mean decrease in accuracy and mean decrease in Gini, are selected (those variables are colored in a different color in Figs 4 to 9).

#### • Tree: Adoption of Digital Banking

**Fig 11:**
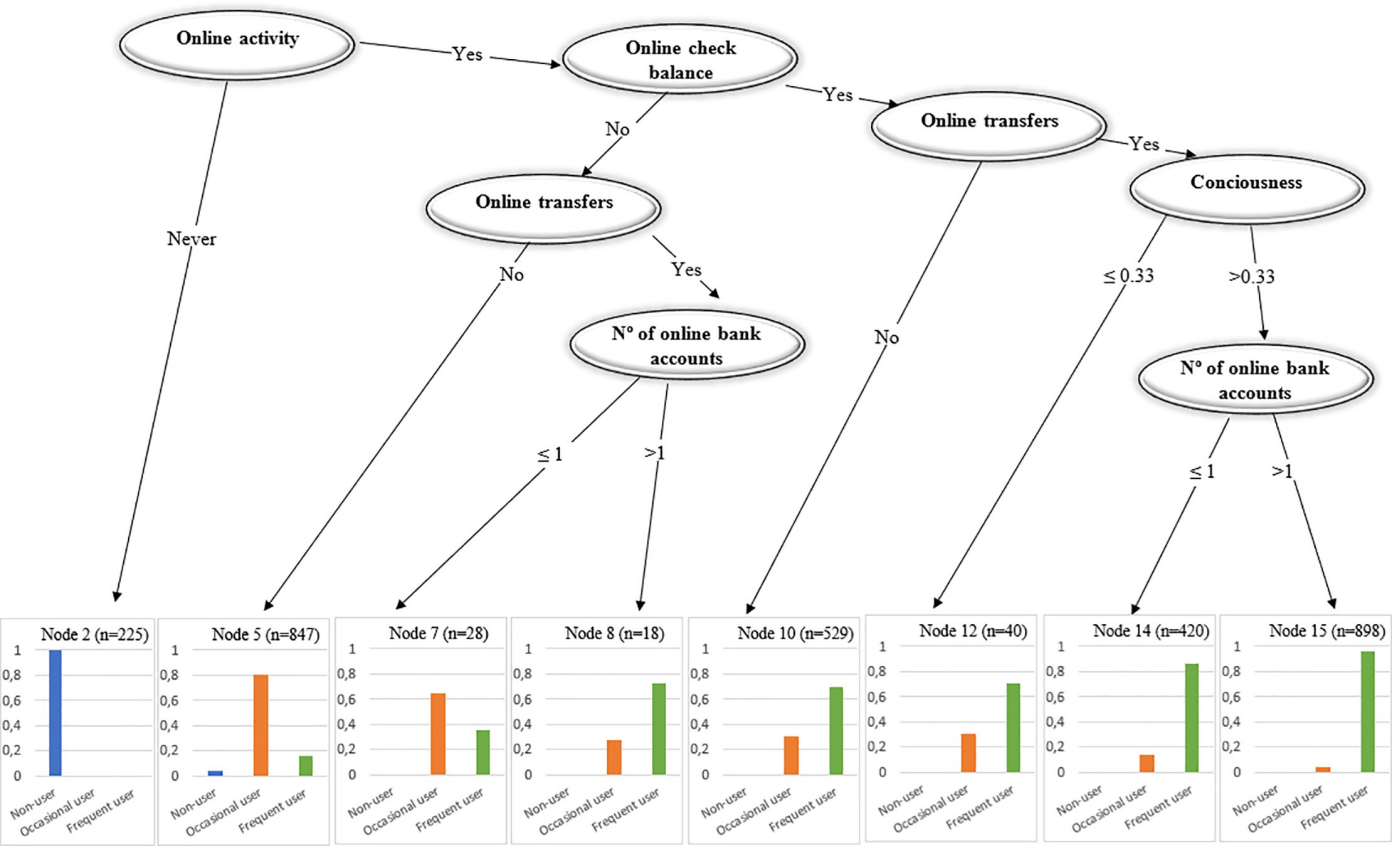
Tree: Adoption of digital banking.

Fig 11 shows that although the range of services available online is wide, the adoption of online banking seems to emerge from customers checking their account balances. It is only after customers check their account balances that they move into transferring money online. Bank customers who do not perform either of these activities are classified as occasional or low frequency users (Node 5). Comparing those individuals who only check their account balances (Node 10) with those who only transfer money (Nodes 7 and 8), checking account balances appears to be more decisive. Furthermore, when customers begin to make transactions and are largely aware of the online possibilities, they become frequent users (Nodes 14 and 15).

An overview of the random models and the classification trees suggests that the main channel by which bank customers become frequent users of online banking services is by their need to check their account balances and, subsequently, transfer money. Consciousness of the availability of online possibilities is also important for the customer to become a frequent digital bank user. Furthermore, the perceived safety of online banking services is not a primary determinant in becoming a frequent user. As we show in the next subsection, safety only becomes influential when customers consider conducting a wide range of transactions online.

#### • Tree: Diversity of Digital Banking Use

**Fig 12:**
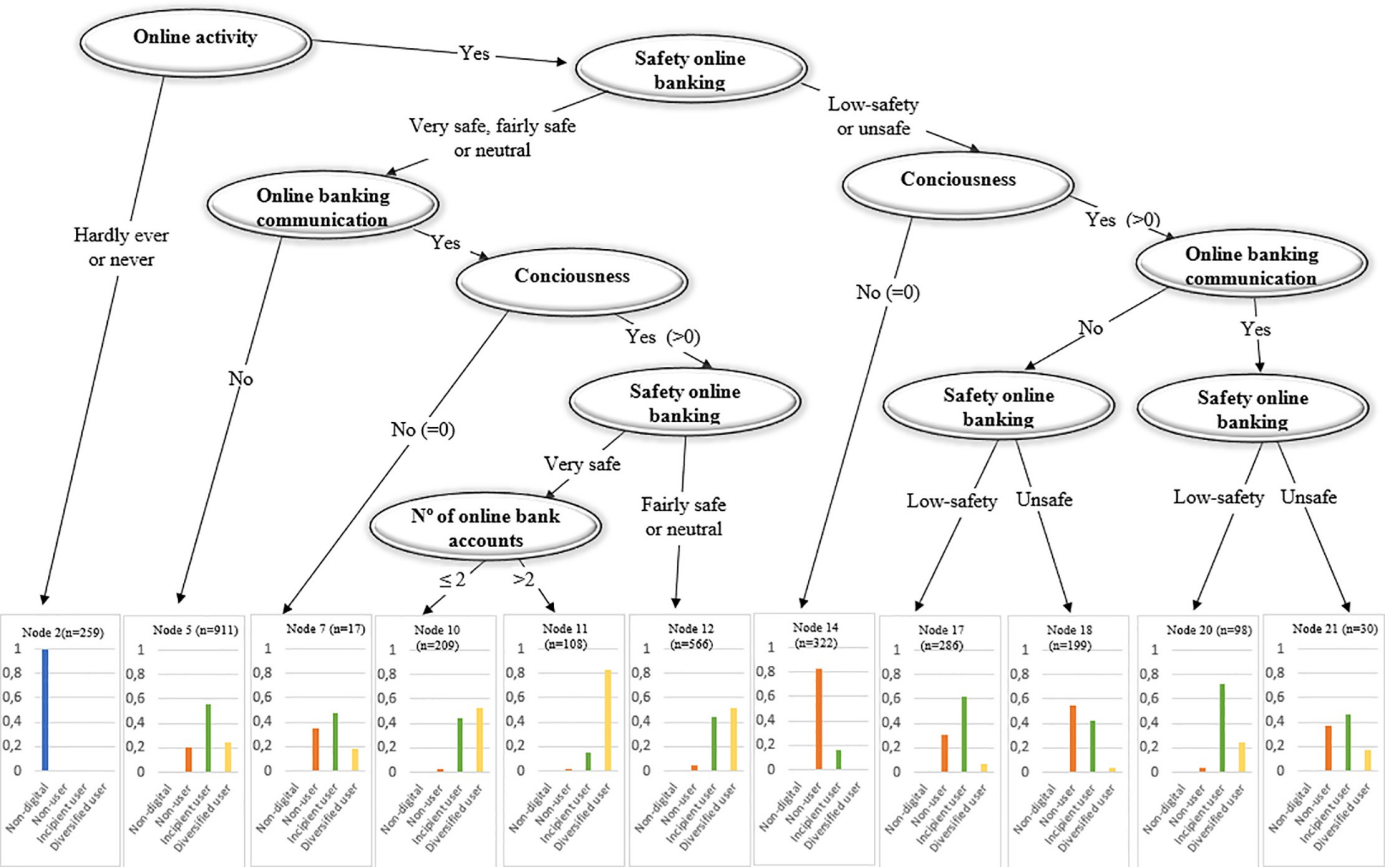
Tree: Diversity of digital use—online banking.

Fig 12 reveals the relevance of the perceived security of online banking in influencing customers’ use of online financial services (Branch 2). Customers who do not consider online banking safe are not likely to become diversified users of online services (Nodes 14–21). Together with safety, customers’ use of digital channels for information purposes and their awareness of the range of online services are key determinants of the diversification of digital services demanded (Node 11). However, consciousness does not compensate for the perceived lack of safety. At most, being conscious make customers switch from non-users to incipient users (Nodes 17–21). Overall, the results suggest that while being a regular online banking user is driven by customers’ needs (e.g., checking account balances and transferring money) as well as by having a certain level of consciousness about the online possibilities, becoming a diversified digital user depends largely on the perceived level of safety.

**Fig 13:**
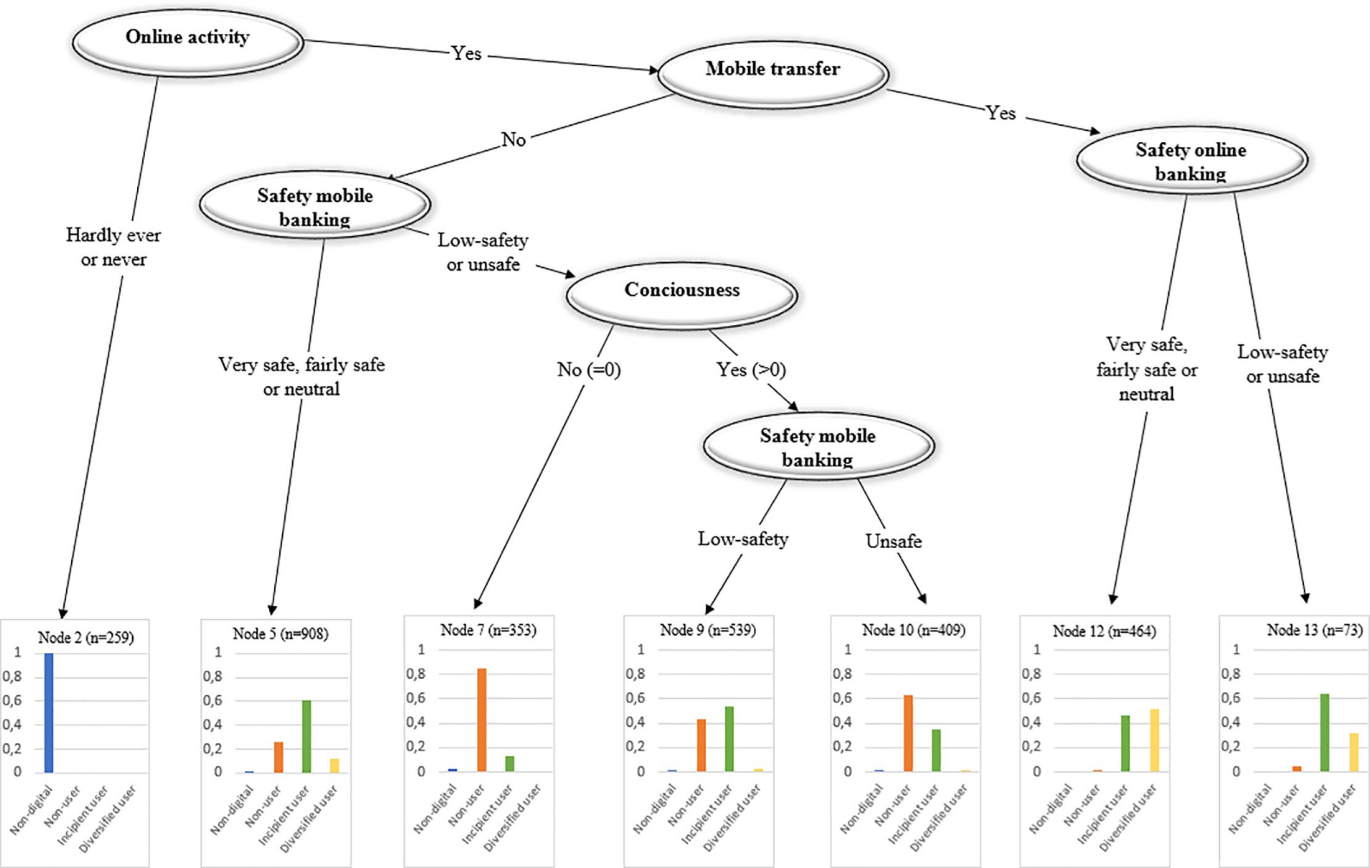
Tree: Diversity of digital use–mobile banking.

Fig 13 plots the classification tree for the diversity of digital use of mobile banking. The results suggest that the diversity of online and mobile banking use are driven by similar factors. The perceived level of safety of mobile banking is also relevant (Node 7). It is unlikely to find diversified users not transferring money with their phones even if they perceive mobile banking as not safe (Node 5).

### • Tree: Adoption of Bank Payment Instruments

**Fig 14:**
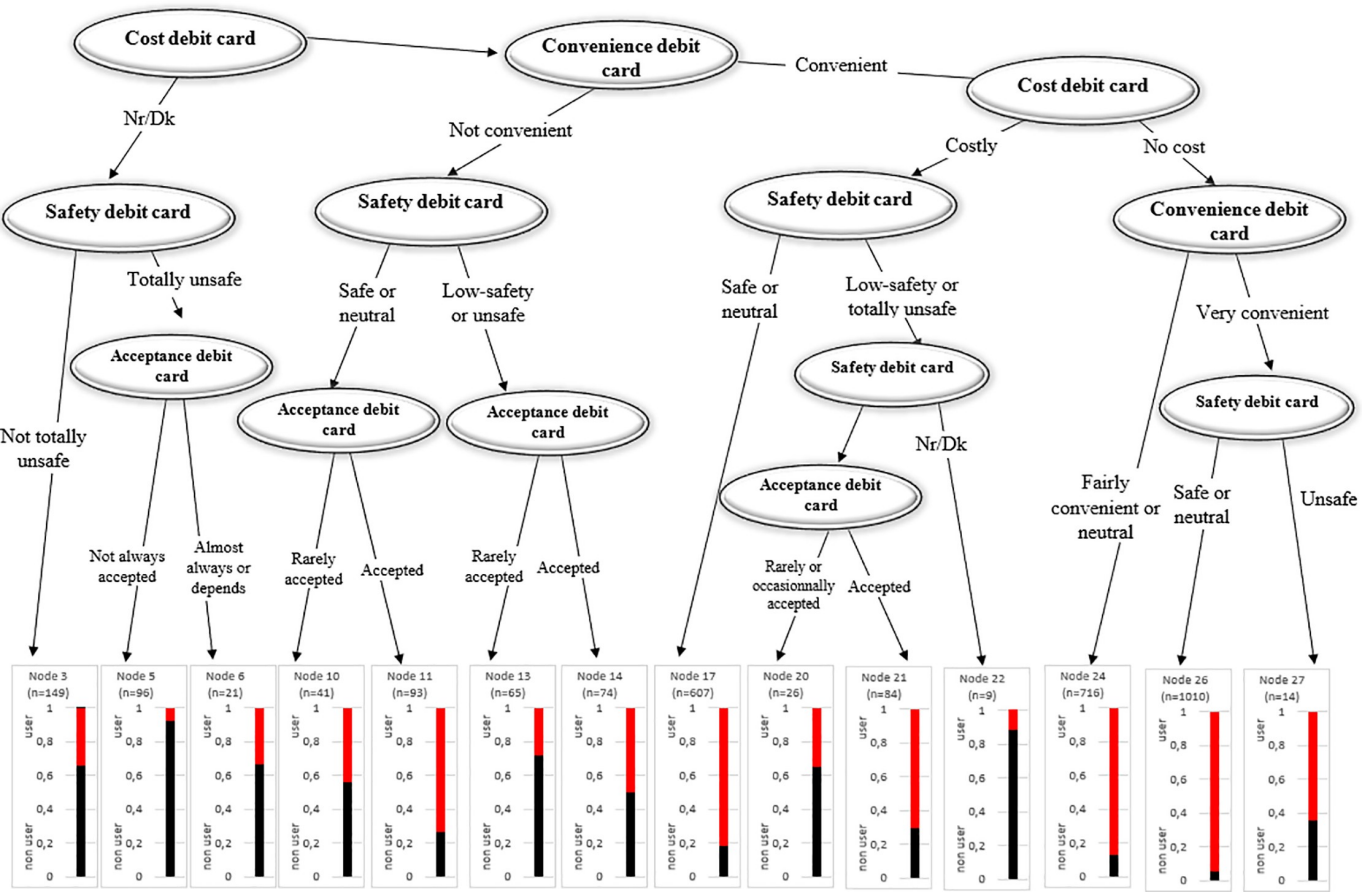
Tree: Debit card use.

**Fig 15:**
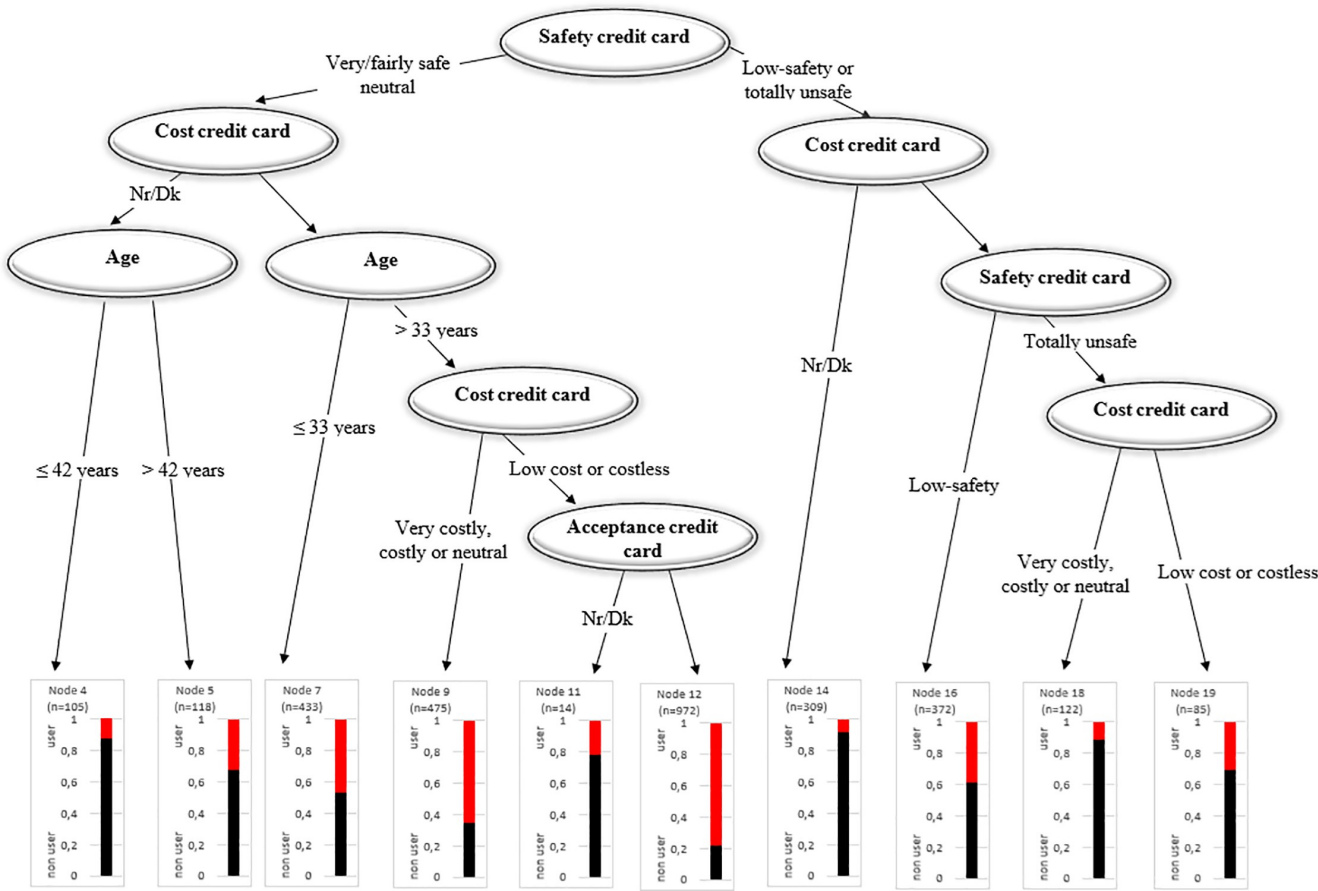
Tree: Credit card use.

Figs 14 and 15 plot the classification trees for debit and credit card adoption, respectively. Both trees demonstrate that safety and cost are the main drivers of adoption. Debit card users can be classified into users who consider debit cards safe, accepted, but not very convenient regardless of their cost (Node 11), and users who consider the method convenient, costless, and safe (Nodes 24 and 26). It can then be argued that a costless perception could compensate for a lack of perceived convenience.

In the case of credit cards, customers who perceive credit cards as unsafe regardless of their cost are less likely to use them (Nodes 14–19). Similar to debit cards, users who perceive credit cards as safe and relatively costless make up the majority of the credit card users (Node 12). The probability of adoption drops to 12% if the credit cards are considered costly.

#### • Tree: Use of Non-Bank Payment Instruments

**Fig 16:**
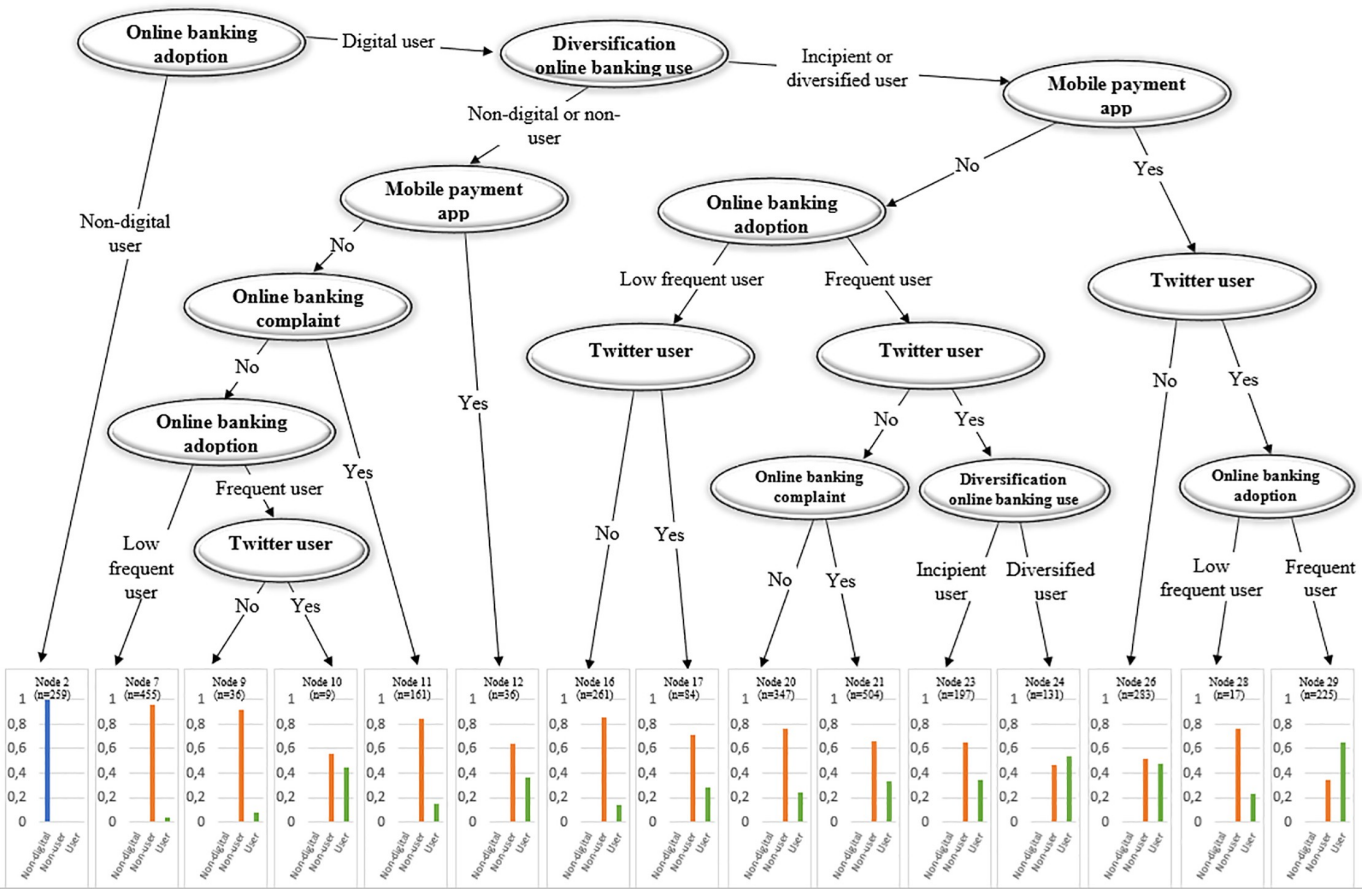
Tree: Use of non-bank payment instruments. Figs 11 to 16 plot the decision trees of bank customer digitalization by estimating a conditional inference tree using those features having the largest predictive power according to the random forest algorithm.

Fig 16 reveals that the adoption of non-bank payment methods occurs when customers are frequent and diversified digital banking users. For occasional and incipient online users, the likelihood of using non-bank payment instruments is quite small. However, as the frequency and diversity of use increases, being active on social media and making mobile payments increases the likelihood that customers would use non-bank payment channels. However, it is worth noting that frequent online users do not use non-bank payment methods if they are just incipient users (Node 23); it is necessary for customers to undertake several digital financial activities to jump into non-bank payments. Similarly, digital banking users who do not have frequent online access are not regular adopters of non-bank payment methods (Nodes 7, 16, 17, and 28).

#### • Robustness: Bayesian network

We also estimate a Bayesian network based on the hill-climbing algorithm, using the subset of features with the largest discriminant power. Bayesian networks could be defined as graphical models of the relationships among a set of variables. These networks use Bayesian inference for probability computations with the aim to model conditional dependence, and therefore causation, by representing conditional dependence by edges in a directed graph [111]. All the graphs are shown in S6 Appendix in S1 File.

Regarding the adoption of online banking, the Bayesian network reveals that checking account balances online and making online transfers are parents of adopting digital banking. Interestingly, the network also reveals that checking account balances online is additionally a parent of making online transfers. This finding suggests that while both kind of activities play a role in the adoption of digital banking services, informational activities (checking account balance) may also foster customers to conduct transactional activities (online transfers). In a way, this result complements our finding that the adoption of digital banking services begins with information-based services (e.g., checking account balance), and is then followed by transactional services (e.g., online/mobile money transfer). Moreover, the Bayesian networks also reveal that effect of being conscious of the range of services that could be conducted online is related to the number of online bank accounts that a customer hold.

Regarding the use of cards as payment methods, it could be observed that the perceived cost of debit and credit cards is a parent of their use. In case of credit cards, the perceived safety is a parent of paying regularly with them. However, for debit cards safety is mediated by customers’ perceptions about cost and convenience. This finding would suggest that while the perceived cost has a direct relationship for both type of cards, it does not seem to be the same in case of the perceived safety. As for the adoption of non-bank payment methods, the network shows that being a diversified digital banking user has a direct relationship on paying with non-bank payment instruments. Additionally, being a Facebook user is a common parent of using non-bank payment instruments, together with being a diversified digital banking user, indicates the presence of interactions between social media and the degree of use online banking in paying with non-bank payment methods.

### 5.5 Causal effects on bank customers’ digitalization: Causal forests

**Fig 17:**
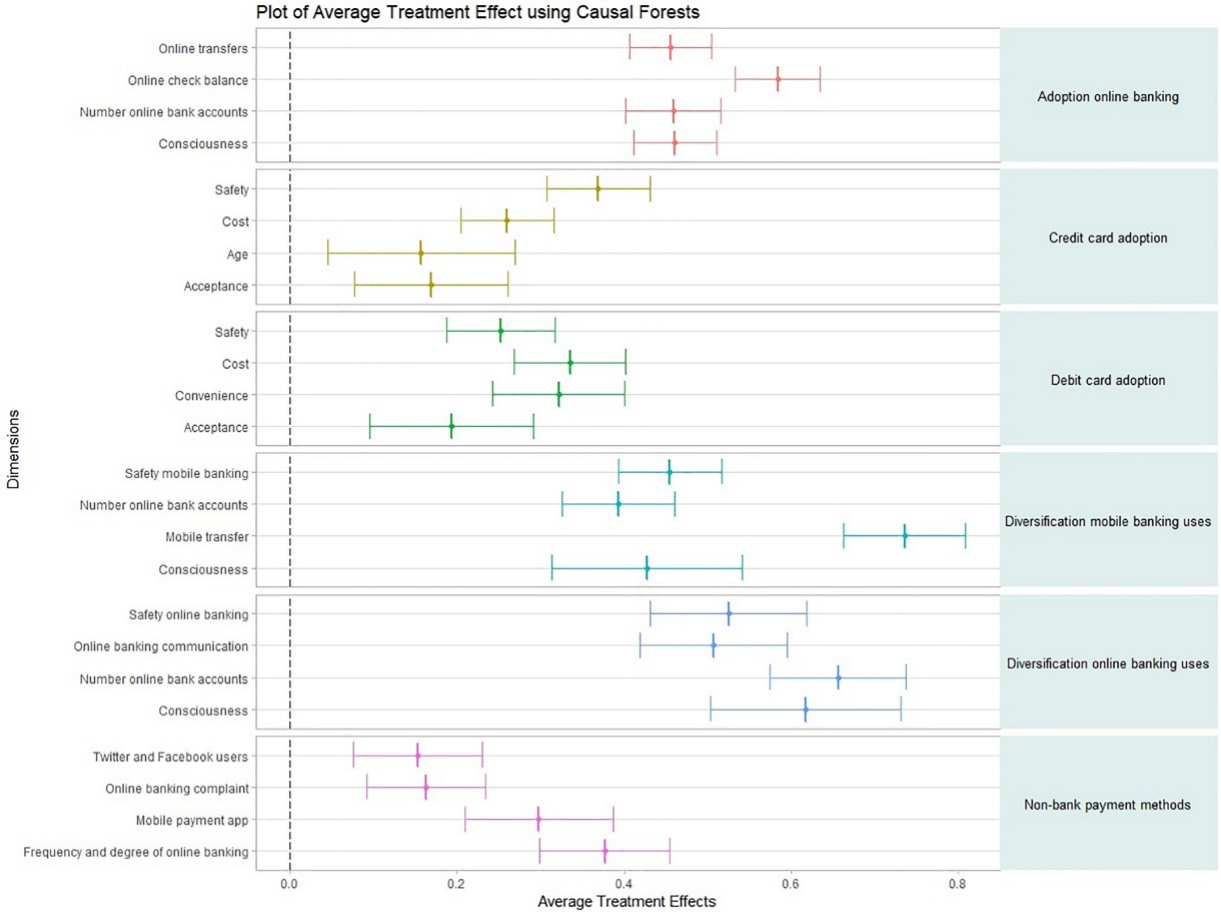
Average treatment effects using causal forests. Fig 17 shows the average treatment effect estimations (ATEs) computed using the causal forest algorithm. The ATEs are shown for each dimension of bamk customers’ digitalization and for those variables with the largest predictive power according to the random forest.

Fig 17 shows the average treatment effect estimations–average differences in the level of digitalization—for those variables identified with the largest predictive power by the random forest. Applying this causal forest algorithm, since the estimated average treatment effects are positive and significantly different from zero, it could be argued that these features drive customers’ levels of digitalization. Then, causal forests reveal that for each of the dimensions examined those features with the largest predictive power also have a large positive effect on the digitalization process. Interestingly, the estimation of the average treatment effects also reveals that checking online balances had the largest effect on adopting online banking while making money transfers with one’s smartphone seems to be relatively more important in order to become a diversified mobile banking customer. Moreover, regarding the use of bank payment methods, we observe that the perception of safety has the largest impact on using credit cards while the perception of cost and convenience have the largest impact on paying with debit cards. This latter result was also highlighted by the Bayesian networks. Finally, regarding non-bank payment methods, the largest effects on adoption come from being a frequent and diversified digital bank customer.

These results confirm that the digitalization of bank customers is largely affected by informational (checking account balances) and transactional activities (online/mobile transfer) while the consciousness about the range of the online services available and the safety of perception have a positive impact on diversifying the use of digital channels.

## 6. Discussion

### 6.1 Supply side explanations

While the variable capturing each customer’s bank does not rank among those with the largest importance, we aim to confirm that the digitalization process is primarily driven by consumers’ characteristics and not by their bank’s characteristics. We then re-run the machine learning algorithm for different samples of consumers aggregated by their main bank characteristics to determine whether or not the predictors and decision trees obtained are qualitatively similar to those obtained in the baseline random forests regressions.

Firstly, since bank size (market power) may play a role in digitalizing customers, we re-run separate regressions for customers of large banks with the largest customer bases in Spain: Santander, BBVA, and CaixaBank. Furthermore, we also conduct a within-bank comparison. This type of analysis helps to ensure that digitalization is not mainly driven by supply-side factors since all the consumers from each subsample would have the same supply level of digitalization. In addition, since the closure of bank branches may force some bank customers to go digital, we also check whether or not bank closures drive digitalization. In doing so, separate regressions are estimated for those customers whose main bank closed at least one branch in their province.

**Fig 18:**
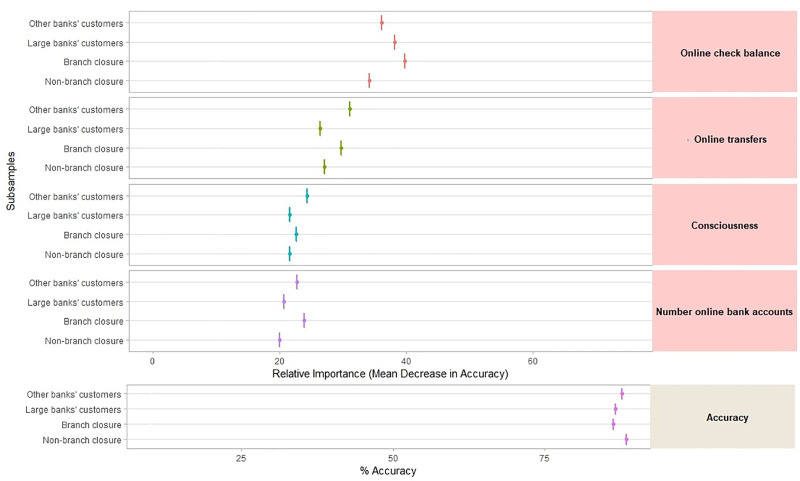
Subsample analysis of supply-side explanations. Fig 18 reports the relative importance—measured by mean decrease in accuracy—of those variables with the largest predictive power for the adoption of online banking by banks’ characteristics: size (large banks’ customers—Santander, BBVA, and CaixaBank—vs other banks’ customers) and branch closure (customers whose main bank closed at least one branch in their province vs customers whose main bank has not closed any branch in their province). The bottom panel shows the predicted accuracy.

Fig 18 reports the relative importance—measured by mean decrease in accuracy—of those variables with the largest predictive power for the adoption of online banking. The full results for the rest of the dimensions are not reported for the sake of simplicity. Checking balances, transferring money, and being conscious of online banking and the number of one’s online accounts are consistently reported as the variables with the largest predictive power across different subsamples. Hence there are not significant differences in the predictive power of the main drivers of adopting of online banking by supply-side factors (banks’ characteristics) nor by the closure of b branches. Similarly, no qualitative differences in the relative importance of the predictors and decision trees obtained are found for other dimensions of digitalization.

**Fig 19:**
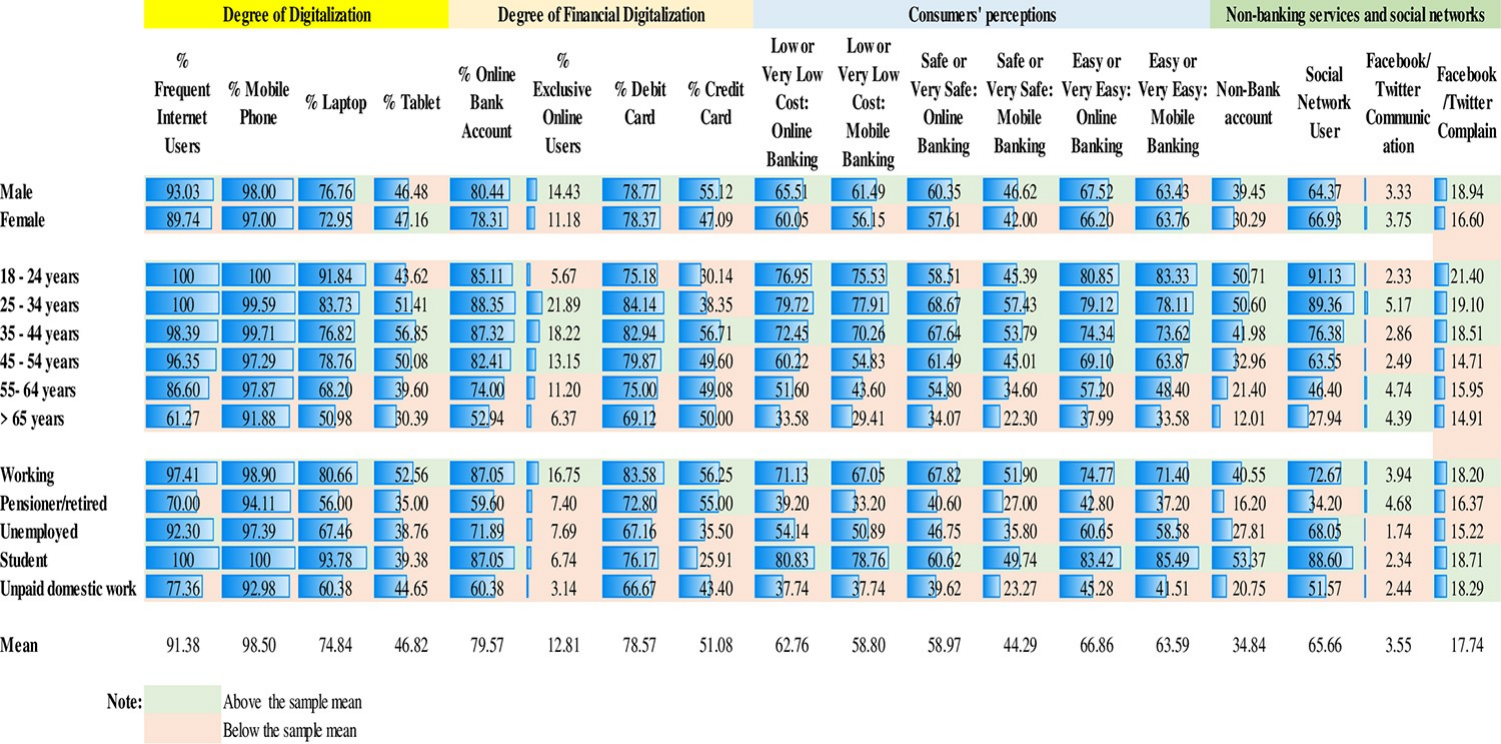
Sample matrix (Heatmap) by dimensions and socio-demographics features. Fig 19 shows the main characteristics of the survey participants by degree of digitalization, degree of financial digitalization, perceptions on mobile and online banking and the use of non-bank services and social networks. Results are presented by gender, age, and employment situation. Each cell represents the percentage of people over the total number of people belong to this category.

These results have interesting business implications as they suggest that the digitalization process is mainly driven by consumer characteristics. This would imply an opportunity for banks to segment customers in order to get on board and retain digital customers. Moreover, the limited impact of the closure of bank branches on digitalization suggests that the digitalization process does not emerge because customers are forced to use digital banking when there are no physical branches to reach out. It seems that customers go digital by their own needs and perceptions not because there are fewer physical branches close to where they live.

### 6.2 Implications, limitations, and scope for future research

Facing digital transformation successfully is among banks’ top priorities. Digital banking is likely to soon become the main channel through which customers interact with their banks. Understanding how customers face the digital jump would help banks to retain their current customers and attract more digital users by, for example, improving those functionalities related to information and transaction-based services. However, since bank customers’ digitalization seems to be explained by the needs and perceptions of consumers, bank marketing strategies should have these dimensions into account. Similarly, the results of the study will help banks understand how their customers could potentially adopt digital payment methods offered by new competitors such as BigTech and FinTech firms.

Just like any other research work, our study has certain limitations. Despite employing is a representative testing ground for research on banking digitalization, it would be ideal to know the digitalization timing of each bank customer in order to provide further insights into the temporal structure of the digitalization process. Our findings are found to be applicable to countries with deep internet penetration, a highly banked population, and a growing use of electronic banking among consumers (e.g. Germany, France, Sweden, United Kingdom, Finland, Italy, United States, Japan, Turkey or Australia). Therefore, it would be interesting to explore whether emerging economies may face the same banking digitalization process documented in this study. It should also be acknowledged that examining bank customers’ digitalization using questionnaire data may involve some biases. In any event, we use a questionnaire that follows the structure of a well-established survey, the Survey of Consumer Payment Choice (SCPC).

Despite these limitations, we believe that the results of this study are valuable for other researchers and practitioners interested in understanding how people go digital. Overall, our study confirms the need to conduct research that covers the entire digitalization process rather than focusing on a single dimension. In addition, our research finds that the application of machine learning techniques on consumer research provides more accurate results that improve the understanding of complex topics.

## 7. Conclusion

Modern societies are undergoing a rapid digital transformation. A sizeable part of this change is related to the demand for financial services. The use of electronic devices such as smartphones, laptops, and tablets to conduct many financial activities has risen sharply. While the banking industry is aware of this transformation, adjusting the supply side depends on related changes in demand.

Understanding the process of financial digitalization is valuable for the banking industry to design strategies that bring on board and retain digital users. It would help banks to obtain information on how they can face competition from new providers of financial services (BigTech and FinTech). Additionally, policymakers may use this knowledge to implement more efficient policies to promote financial digitalization and enhance financial inclusion and literacy. To reach this end, this paper employs a machine learning approach to reveal the patterns driving the digitalization process and to offer a multi-dimensional comprehensive picture of the process by which bank customers become digitalized. While most previous studies have discussed the determinants of certain adoption decisions, we outline the sequence of steps that customers follow to adopt digital financial services and become diversified users. Several dimensions are considered: adoption of online banking, diversification of the use of online services, and the choice of bank versus non-bank payment instruments. Our approach benefits from the advantages of machine learning techniques, including the capacity to identify complex and nonobvious patterns or knowledge hidden in a database with millions of data points. These techniques are applied to an in-depth consumer survey specifically designed for the purpose of this study. Furthermore, we run causal forest models to examine the causal relationships on the digitalization process.

The empirical results suggest that the digitalization process is originated from customers’ need to gain information about basic aspects of their banking accounts (e.g., checking their account balances), and this facilitates a transition to transactional services (e.g., transferring money). We also find that once the initial adoption has taken place, the diversification of online and mobile services adopted by the customers becomes larger when they are conscious of the range of possibilities provided by the bank and when they perceive those options as safe. Taken together, these results suggest that while customers’ perceptions are important on using digital channels, in banking the adoption is primarily driven by information-based services. Furthermore, we show that the adoption of non-bank payment instruments (e.g., PayPal and Amazon) happens when consumers are already diversified digital bank customers. Users of non-bank payment instruments seem to have previously reached a substantial degree of banking digitalization. This suggests a certain degree of complementarity between bank and non-bank digital services.

The causal machine algorithm reveals that among the information-based activities, checking online balances has the largest effect on adopting online banking. Similarly, making money transfers with a smartphone is the transactional-based activity that is relatively more important to define a diversified mobile banking customer. These results are confirmed by Bayesian networks, which also indicate that the relevance of interactions between social media and the degree of use online banking and non-bank payment methods. Importantly, we find that the digitalization process is not mainly driven by bank characteristics. We report a limited impact of the closure of bank branches on digitalization, which suggests that customers go digital by their own needs and perceptions not because there are fewer physical branches close to where they live (a diminishing role of geographic distance in banking).

These findings are relevant to better understand the digital transformation of consumers. While prior theories and studies have given prominence to the technological components of the service and to consumers’ perceptions to explain the digital jump, our machine learning approach reveals that customers go digital first for information-based needs and, later, to undertake transactional services.

Overall, the findings of the study suggest that financial providers could benefit from the digitalization phenomenon by offering services that better match customers’ needs. In this sense, segmenting customers using similar techniques and data, would make possible to offer them more personalized digital services. Moreover, linking payments experiences to social media interactions could also be used to foster the adoption of digital payments. Finally, our findings could be used by policymakers to improve the communication and social awareness of the range of online services available, as part of the policies and official strategies to promote financial digitalization.

## Supporting information

S1 File (PDF)
