# Supplementary material for: A machine learning approach to the digitalization of bank customers: Evidence from random and causal forests
Source: PLoS One. 2020 Oct 28;15(10):e0240362. doi: 10.1371/journal.pone.0240362 (PMC7593085; doi:10.1371/journal.pone.0240362)
Supplement: S1 File — (PDF) [file pone.0240362.s001.pdf]

Supporting information

S1 Appendix. Sample Design and Data Collection Process

Sample design	Landline	• Phase 1: the municipality. Stratified randomized selection using the size of the municipality and the region.<br>• Phase 2: household. Randomized selection using the Irismedia directory recoded and debugged by IMOP.<br>• Phase 3: individual. Selection employing sex and age quotas.<br>The application selects the household member who is relatively less represented in the sample at the time of the call and establishes a postponement if the chosen person is not at home at that moment.
	Mobile phone	Simple random selection using the mobile phone database generated by IMOP from the data provided by each mobile operator. This database was tested before beginning the survey in order to detect inactive lines.
Technique		All the interviews are conducted through the CATI system using a computer.
Sample error		±1.8% for a confidence level of 95.5%.
% mobile phone interviews		40.4%
% landline (fixed phone) interviews		59.6%
Questionnaire duration (on average)		21.5 minutes
Denial rate		14.7% of the people who took the telephone call declined to answer the questionnaire.
Not completion		0.9% of the people who began answering the questionnaire decided to end the survey before its completion.
Data availability		All data are available at Funcas Foundation (Caballero de Gracia, 28 28013 Madrid). Contact details: odf@funcas.es

S2 Appendix. List of Survey Questionnaire Variables

Socio-demographic characteristics	Age	Financial status	Nº bank accounts	Financial activities	Online/Mobile check balance	Cash	Home
	Gender		Nº banks		Online/Mobile communication		Pocket
	Province		Savings bank account		Online/Mobile pay bills		Annual cash payments
	Employed worker		Current bank account		Mobile web browser	Non-bank user	Nonbank payment user
	Employment situation		Nº Online bank accounts		Mobile purchase		Google Wallet
	Sector activity		Nº Online only bank acc.		In-app purchase		Amazon payments
	Unemployment period		Consciousness		QR code		Paypal
	Full-time job		Main bank		SMS		Web account
	Permanent job contract		Credit/Debit card holder		Wave mobile		Other
	Monthly revenue	How many times do you . . .?	Nº bank branch check		Online bank communication	Where do you get cash?	ATM cash
	Household employees		Nº check credit weekly		Online bank complaint		Bank branch cash
	Household monthly rev.		Nº check monthly bank acc.		Phone complaint		Family cash
	Workplace province		Nº check monthly credit	How often do you . . .?	Freq bank branch check		Cashback cash
Social profile	Facebook user		Nº check prepaid		Freq check bank account		Never cash
	Twitter user		Nº check prepaid weekly		Freq check credit		Nº where cash
	Twitter/Facebook bank communication		Nº check weekly bank acc.		Freq check prepaid		Times withdrawal
	Twitter/Facebook bank complaint		Nº check weekly credit		Freq online	Been	Victim fraud
Responsible for?	Household financial		Bank branch check		Freq online check		
	Monthly bills		Check monthly prepaid		Freq phone complaint	Digital media	Landline or mobile
	Household savings		Check weekly prepaid		Freq use online		Tablet
	Household shopping		Nº check weekly credit		Freq withdrawal		Computer or laptop
							Workplace connection

Customers’ perceptions

Customers’	Acceptance	ATM withdrawal, bank account number, credit card, debit card, mobile banking, online banking, prepaid card
	Convenience	
	Cost	
	Difficulty	
	Easiness	
	Quality	
	Safety	
	Risk pay	App, email, online, personally, SMS
	Value	Confidentiality, easiness, protect. losses, speed deduction, speed payment, speed registration

S3 Appendix. Dictionary

Dimension	Classification	Definition
Adoption of Digital Banking	Non users	Individuals who over the course of the year have not adopted any kind of financial digitalization, including those who are not even digitalized consumers (i.e., they do not use the internet)
	Occasional users	Individuals who conducted online banking activities, but not on a monthly basis.
	Frequent users	Individuals who conducted online financial activities every month over the course of the year.
Degree of use of Digital Banking	No digital users	Individuals who are outside of the digitalization process (i.e., who have no access to the internet)
	Non-users of digital financial services	Individuals who are frequent internet users but do not conduct any financial activity online
	Incipient users	Individuals who perform some but not all online financial activities at least once a month.
	Diversified users	Individuals that carry out all financial activities online at least once a month.
Adoption of Banks' Payment Instruments	Non-debit card users	Individuals who do not use on a monthly basis a debit card to make payments.
	Debit card users	Individuals who use on a monthly basis a debit card to make payments.
	Non-credit card users	Individuals who do not use on a monthly basis a credit card to make payments.
	Credit card users	Individuals who use on a monthly basis a credit card to make payments.
Adoption of Non-Bank Payment Instruments	No digital users	Individuals who are outside of the digitalization process (i.e., who have no access to the internet).
	Non-users of non-banking payment methods	Individuals who do not use at least once a month a payment method which is provided by a non-bank institution (e.g. Amazon Pay, PayPal, Google Wallet, Apple Pay, etc.) were classified as non-users of non-bank payment instruments.
	Users of non-banking payment methods	Individuals who use at least once a month a payment method which is provided by a non-bank institution (e.g. Amazon Pay, PayPal, Google Wallet, Apple Pay, etc.) were classified as non-users of non-bank payment instruments.

S4 Appendix. Alternative models performance in terms of predictive accuracy

		Out-of-sample accuracy (70/30% split)					
		Adoption of online banking	Diversity of digital use: online banking	Diversity of digital use: mobile banking	Debit card	Credit card	Adoption of Non-bank payment methods
**Random forest**		88.41%	70.11%	70.01%	85.00%	74.89%	76.14%
**Extreme Gradient Boosting**		84.99%	68.82%	67.85%	84.79%	73.51%	75.91%
**K-Nearest Neighbor**	**Euclidean**	84.92%	63.41%	63.97%	80.60%	64.75%	74.94%
	**Manhattan**	82.71%	60.53%	62.97%	80.27%	65.96%	72.73%
	**Chebyshev**	65.85%	50.55%	50.67%	79.71%	58.76%	66.19%
**Supportive Vector Machine (SVM)**	**Linear**	83.54%	69.00%	66.70%	82.75%	71.14%	74.15%
	**Radial**	84.58%	67.36%	66.27%	82.11%	72.63%	74.48%
	**Sigmoid**	83.43%	68.89%	66.16%	79.23%	65.60%	73.49%
	**Polynomial**	80.32%	58.60%	61.56%	79.98%	67.84%	72.95%
**Bayesian Networks**	**Naive Bayes**	58.52%	39.55%	43.52%	67.16%	56.25%	53.30%
	**Tan HSCP**	58.98%	41.48%	43.18%	65.91%	55.68%	52.61%
	**Tan CL**	34.55%	25.34%	28.64%	53.86%	49.09%	35.23%
	**Tan HC**	57.39%	40.45%	41.02%	65.57%	57.95%	52.16%
	**AODE**	58.07%	42.39%	42.95%	65.68%	57.50%	53.86%
	**KBD**	56.82%	39.89%	44.66%	66.70%	58.30%	51.59%
	**FSSJ**	86.48%	66.36%	63.98%	84.43%	72.16%	70.91%
	**BESJ**	81.14%	66.00%	60.91%	75.00%	64.55%	66.93%
**Artificial Neural Networks: Extreme learning machine**	**Sigmoid**	58.48%	40.24%	45.12%	79.82%	54.55%	64.86%
	**Radial Basis**	41.22%	28.60%	29.49%	59.20%	48.78%	45.68%
	**Sine**	37.65%	26.50%	30.27%	52.99%	51.77%	37.92%
	**Hard-Limit**	56.29%	39.58%	44.24%	79.82%	54.10%	64.86%
	**Symm. Hard-Limit**	58.48%	39.91%	44.35%	78.38%	54.32%	61.31%
	**Satlins**	73.49%	60.31%	61.86%	82.59%	69.73%	73.17%
	**Tan-Sigmoid**	58.48%	40.24%	45.79%	77.83%	54.21%	64.86%
	**Triangular Basis**	82.18%	65.63%	67.96%	84.15%	74.61%	76.05%
	**Rectifier Linear Unit**	82.18%	65.41%	61.75%	83.26%	74.84%	75.84%
	**Linear Function**	82.44%	65.96%	67.74%	84.04%	74.72%	75.94%
**Logit**		79.27%	55.01%	59.57%	84.23%	70.62%	73.46%

S4 Appendix reports the predicted accuracy for all the models (machine learning algorithm and logit) employed in examining the digitalization of bank customers.

S5 Appendix. Features importance by the Extreme Gradient Booting algorithm

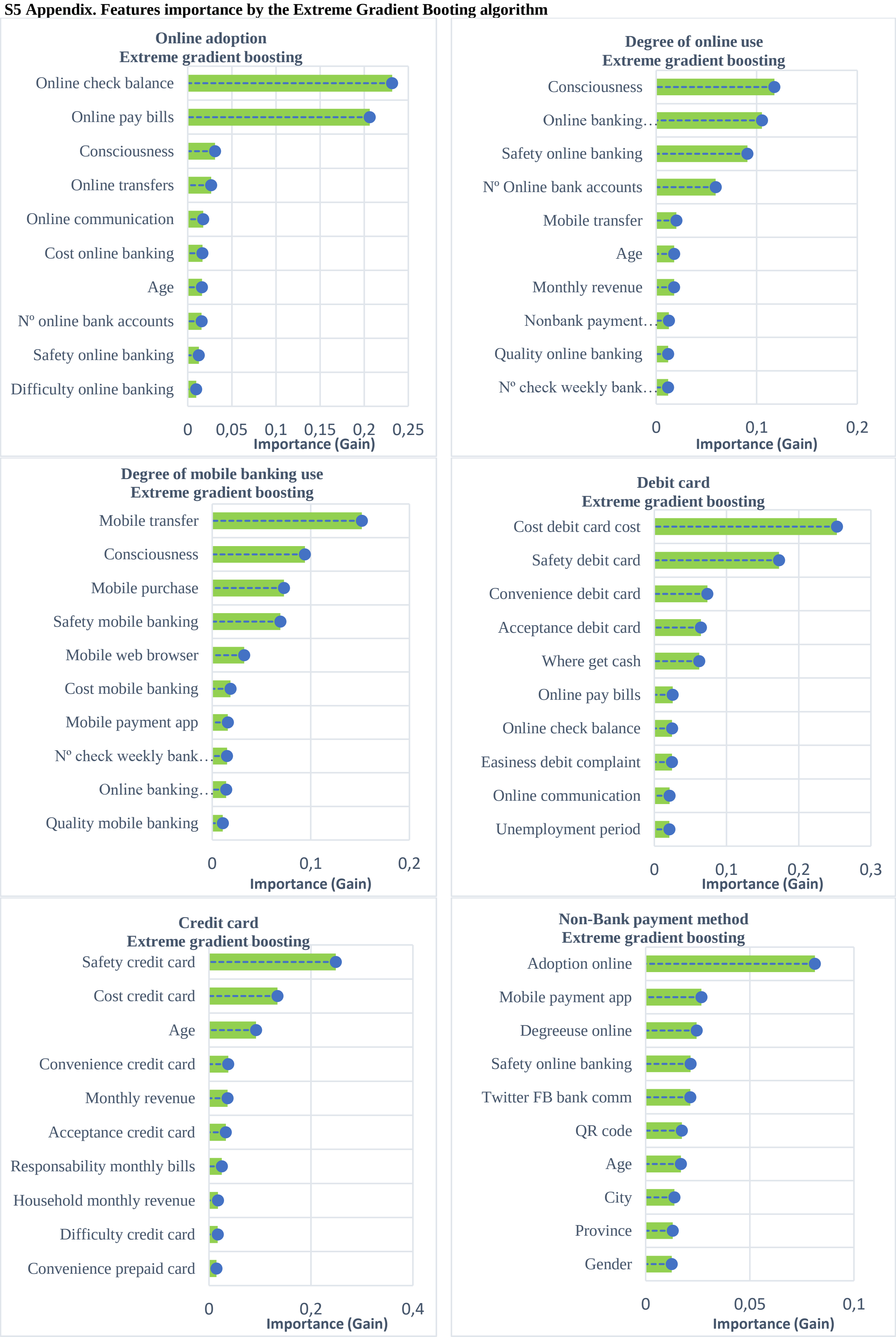

These figures provide the most important features predicting bank customers' digitalization based on the Extreme gradient boosting algorithm. The relative importance of each feature is computed using the relative contribution of the corresponding feature to the model calculated by taking each feature's contribution for each tree in the model (Gain). A higher score suggests the feature is more important in the boosted tree prediction.

S6 Appendix. Bayesian Networks.

Figure S6.1 Bayesian Network: Adoption of digital banking

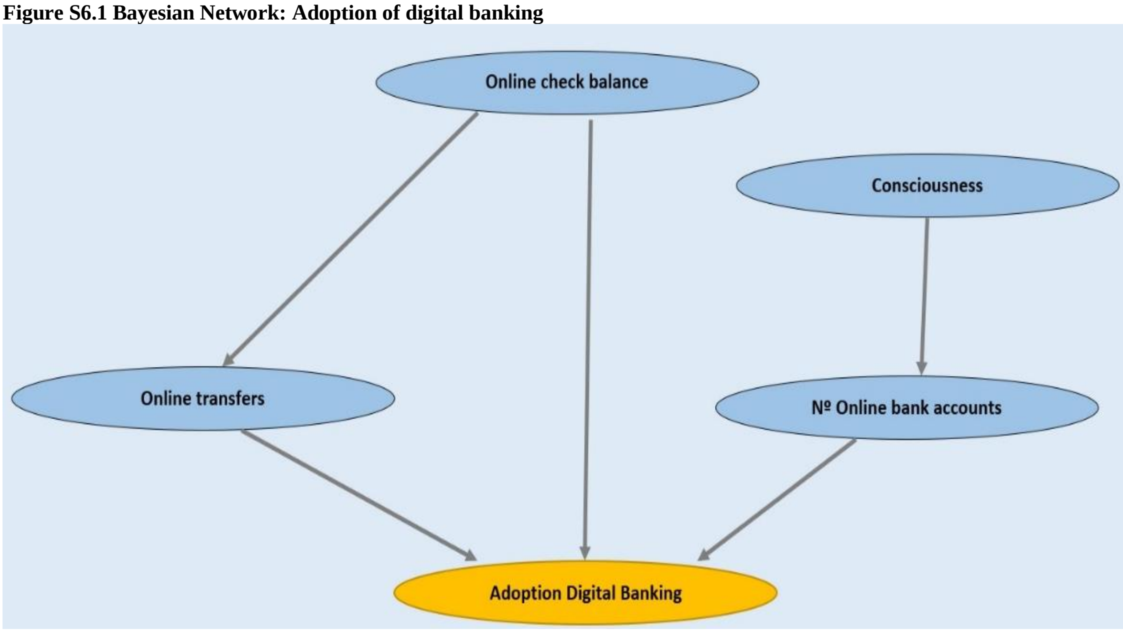

Figure S6.2 Bayesian Network: Diversity of use - Online banking

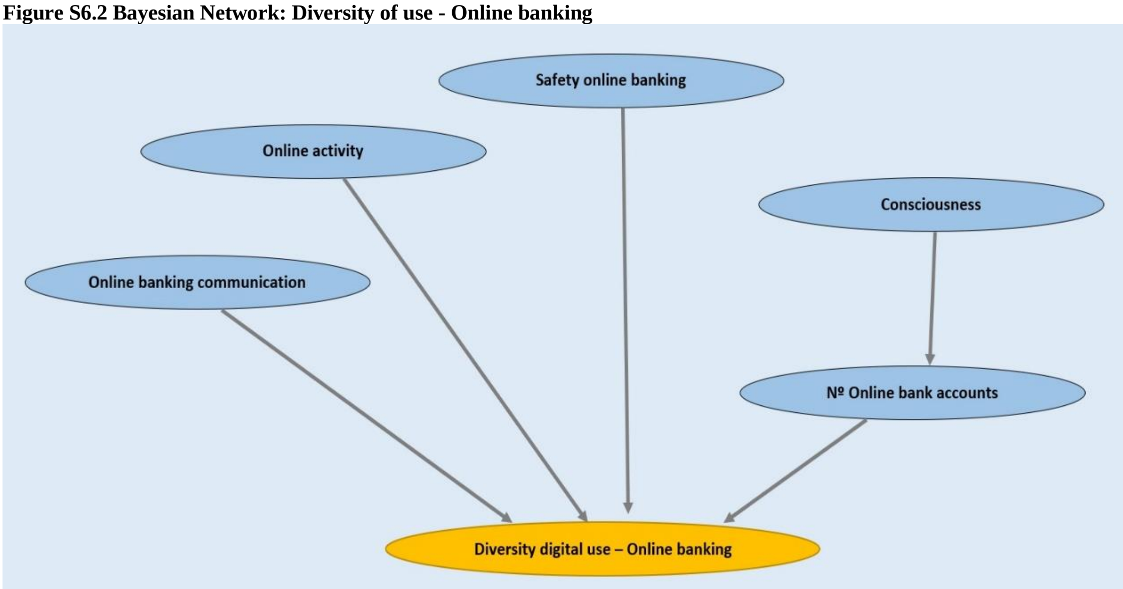

Figure S6.3 Bayesian Network: Diversity of use - Mobile banking

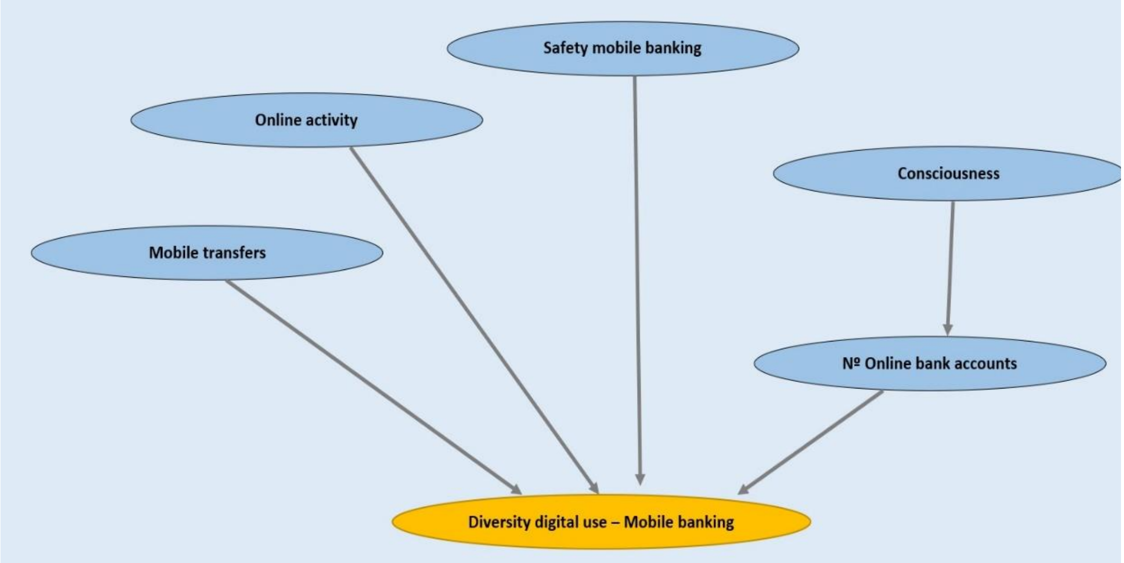

Figure S6.4 Bayesian Network: Debit card use

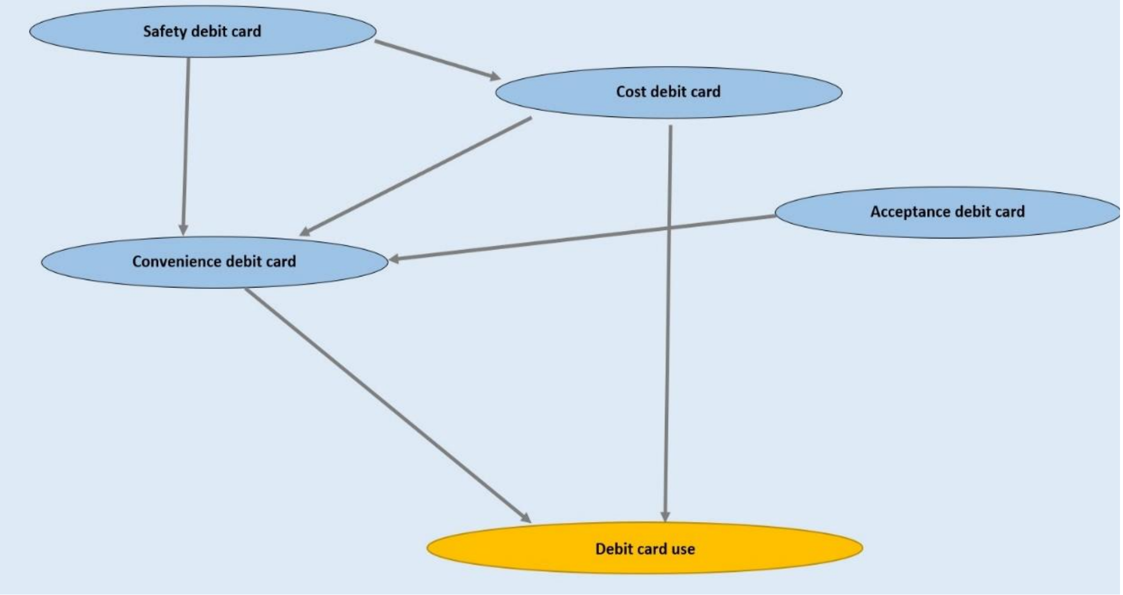

Figure S6.5 Bayesian Network: Credit card use

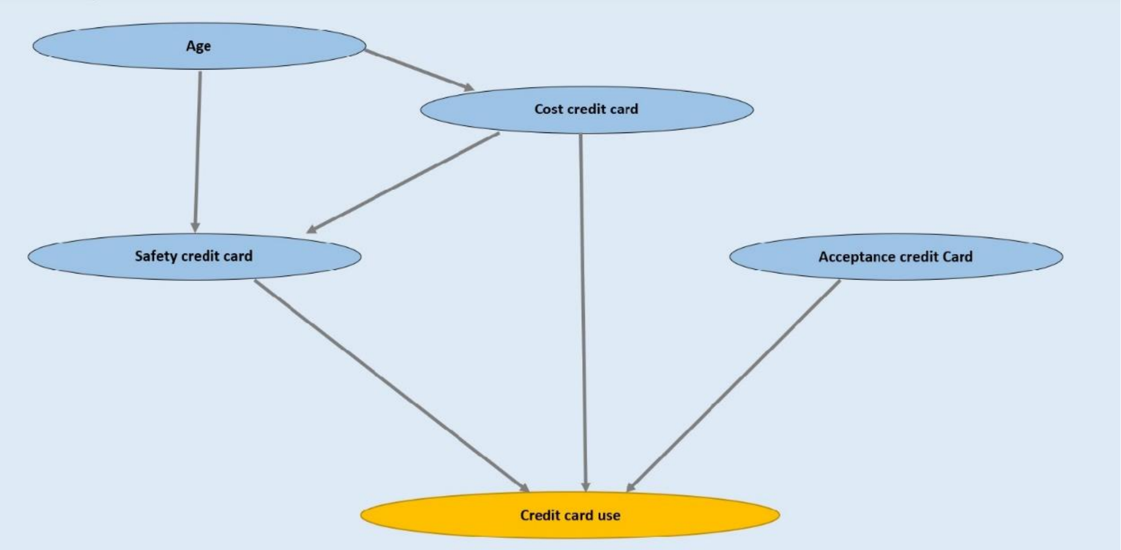

Figure S6.5 Bayesian Network: Use of non-bank payment instruments

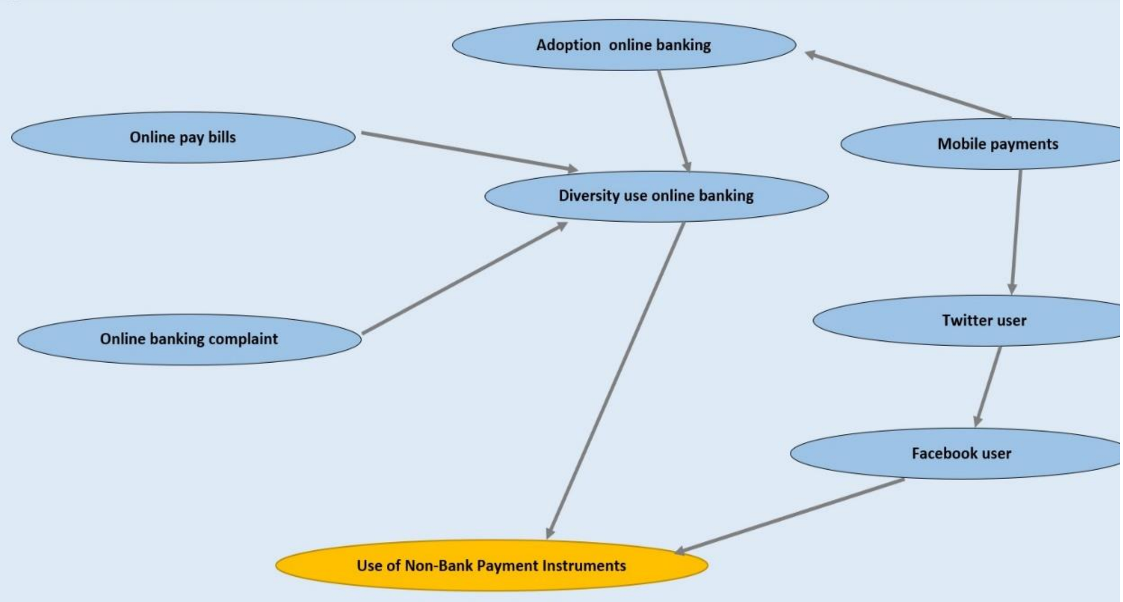

These figures plot the Bayesian network, based on the hill-climbing algorithm, for the subset of features with the largest discriminant power for each of the dimensions considered.
